# Comparison of the integrin α4β7 expression pattern of memory T cell subsets in HIV infection and ulcerative colitis

**DOI:** 10.1371/journal.pone.0220008

**Published:** 2019-07-29

**Authors:** Melanie Wittner, Veronika Schlicker, Jana Libera, Jan-Hendrik Bockmann, Thomas Horvatits, Oliver Seiz, Silke Kummer, Carolin F. Manthey, Anja Hüfner, Marcus Kantowski, Thomas Rösch, Olaf Degen, Samuel Huber, Johanna M. Eberhard, Julian Schulze zur Wiesch

**Affiliations:** 1 I. Department of Medicine, University Medical Center Hamburg-Eppendorf, Hamburg, Germany; 2 Infectious Disease Unit, University Medical Center Hamburg-Eppendorf, Hamburg, Germany; 3 German Center for Infection Research (DZIF), partner site Hamburg, Lübeck, Borstel, Riems, Germany; 4 Clinic and Polyclinic for Interdisciplinary Endoscopy, University Medical Center Hamburg-Eppendorf, Hamburg, Germany; Emory University School of Medicine, UNITED STATES

## Abstract

Anti-α4β7 therapy with vedolizumab (VDZ) has been suggested as possible immune intervention in HIV. Relatively little is known about the α4β7-integrin (α4β7) expression of different T-cell subsets in different anatomical compartments of healthy individuals, patients with HIV or inflammatory bowel disease (IBD). Surface expression of α4β7 as well as the frequency of activation, homing and exhaustion markers of T cells were assessed by multicolour flow cytometry in healthy volunteers (n = 15) compared to HIV infected patients (n = 52) or patients diagnosed with ulcerative colitis (UC) (n = 14), 6 of whom treated with vedolizumab. In addition, lymph nodal cells (n = 6), gut-derived cells of healthy volunteers (n = 5) and patients with UC (n = 6) were analysed. Additionally, we studied longitudinal PBMC samples of an HIV patient who was treated with vedolizumab for concomitant UC. Overall, only minor variations of the frequency of α4β7 on total CD4^+^ T cells were detectable regardless of the disease status or (VDZ) treatment status in peripheral blood and the studied tissues. Peripheral α4β7^+^ CD4^+^ T cells of healthy individuals and patients with UC showed a higher activation status and were more frequently CCR5^+^ than their α4β7^-^ counterparts. Also, the frequency of α4β7^+^ cells was significantly lower in peripheral blood CD4^+^ effector memory T cells of HIV-infected compared to healthy individuals and this reduced frequency did not recover in HIV patients on ART. Conversely, the frequency of peripheral blood naïve α4β7^+^ CD4^+^ T cells was significantly reduced under VDZ treatment. The results of the current study will contribute to the understanding of the dynamics of α4β7 expression pattern on T cells in HIV and UC and will be useful for future studies investigating VDZ as possible HIV cure strategy.

## Introduction

α4β7 is an integrin which is expressed on different circulating lymphocytes and which interacts with MAdCAM-1 expressed on venules within different gastrointestinal tissues (GIT) and thus facilitates homing of α4β7^+^ cells to the gut [1]. Importantly, the gut and the gut-associated lymphoid tissue (GALT) are critical sites of HIV replication and CD4^+^ T-cell depletion [2–5]. Several studies have reported that α4β7^high^ CD4^+^ T cells are highly susceptible to HIV and SIV infection, and are preferentially depleted in the blood and the gut during primary HIV infection [6–9]. Remarkably, HIV particles were demonstrated to transport α4β7 on their surface after budding, aiding homing of the virions to the GIT [10]. Even the acquisition and progression of HIV can be predicted by the α4β7 expression of peripheral CD4^+^ T cells [6]. In a landmark study conducted in macaques it could be shown that antiretroviral treatment (ART) and subsequent administration of an α4β7-specific antibody led to virologic control of SIV for up to 50 weeks after the withdrawal of both antibody and ART [11].

A similar therapeutic antibody (vedolizumab/Entyvio) has been approved for the treatment of inflammatory bowel diseases (IBD, i.e. Crohn’s disease/ulcerative colitis) several years ago. Currently, several therapeutic vedolizumab trials that explore different HIV cure strategies are under way (2018-000497-30, 2017-003081-27, NCT02788175, NCT02972450).

However, it is controversial whether the therapeutic effect of vedolizumab seen in SIV can be replicated in HIV-1 infection [12]. A recent study by Uzzan *et al*. illustrated the link between anti-α4β7 therapy and possible targeting of the HIV viral reservoir in the gut [13]. In this study it was demonstrated that the administration of an anti-α4β7 antibody in patients with mild inflammatory bowel disease and concomitant HIV infection led to an attenuation of lymphoid aggregates in the terminal ileum. However, few studies have analysed the α4β7 expression pattern of T cells in different peripheral and gut-resident T-cell subsets of healthy individuals, patients with inflammatory bowel disease after the administration of vedolizumab or HIV patients in greater detail [14–17].

Here, we further define the distribution of α4β7 on different T-cell subsets and their composition in peripheral blood, lymph nodes and the gut mucosa of HIV infected patients versus healthy volunteers and patients with IBD, some of them treated with vedolizumab.

## Materials and methods

### Study subjects, sample acquisition and processing

Cryoconserved peripheral blood mononuclear cells (PBMC) were isolated and used for immunophenotypic staining as previously described [18,19]. Gut biopsies were obtained during routine coloscopies. Five double biopsies from the sigmoid colon mucosa were drawn into sterile PBS and processed directly as previously described by Morón-López *et al*. with minimal adaptations [20]. In brief, the tissue was disintegrated by short digestion with Hank's Balanced Salt Solution (HBSS) containing DTT and EDTA before samples were incubated in 6-well, low-binding plates in HBSS supplemented with 10% FCS, antibiotics and antifungals overnight (1 mg/mL Piperacillin/Tazobactam and 1,25 μg/mL Amphotericin B). The next day, the remaining tissue was disrupted by pipetting and mononuclear cells (lamina propria lymphocytes, LPL) were collected from the supernatant and were stained and measured directly.

Lymph nodal mononuclear cells (LNMC) were processed and thawed as previously described (11). Written informed consent was obtained from all participants who were recruited for this study at the University Medical Center Hamburg-Eppendorf. The study was approved by the local Institutional Review Board of the Ärztekammer Hamburg (MC-316/14, PV4444, PV4870, PV5798) and conducted in accordance with the Declaration of Helsinki. Clinical and demographic information like CD4^+^ T-cell counts, plasma viral loads, disease activity of the ulcerative colitis [21], current medication or treatment history were extracted from the clinical database. For an overview of processed samples and numbers, see **Tables 1 and 2 and S1 Table.**

**Table 1:** Cohort statistics of HIV-positive and healthy individuals.

Classification	N	Age	M/F (% M)	Viral load (Copies/mL)	CD4 count (Cells/μL)
Healthy subjects	15	28 (19–43)	7/8 (47%)	n.a.	n.a.
HIV patients on ART	23	29 (46–71)	20/3 (87%)	n.a.	388 (109–1030)
Viremic HIV patients	24	43 (20–67)	19/5 (79%)	178 000 (41 500–5 300 000)	316 (6–829)
HIV elite controllers	5	37 (21–71)	4/1 (20%)	n.a.	994 (375–1219)
LN of HIV-infected patients	3	38 (37–38)	3/0 (100%)	379 288 (8576–750 000)	465 (265–665)
LN of uninfected individuals	3	48 (28–53)	2/1 (67%)	n.a.	n.a.

PBMC were collected. Values are medians (ranges). *Level of quantification: 50 copies/mL. LN, lymph node; M, male; F, female.

**Table 2:** Overview over processed samples and conducted experiments.

Samples	N	Kind	Antibody (Combination)	Figure
Healthy subjects	15	PBMC	α4β7-specific (clone Act1)	2, 3, S9
HIV patients on ART	23	PBMC	α4β7-specific (clone Act1)	2, 3, S9
Viremic HIV patients	24	PBMC	α4β7-specific (clone Act1)	2,3, S9
HIV elite controllers	5	PBMC	α4β7-specific (clone Act1)	2,3
Healthy subjects	9	PBMC	α4-specific antibody (clone 7.2R) + β7-specific antibody (clone FIB504)	4,5, S10
Ulcerative colitis baseline	8	PBMC	α4-specific antibody (clone 7.2R) + β7-specific antibody (clone FIB504)	4,5, S10
Ulcerative colitis + VDZ	6	PBMC	α4-specific antibody (clone 7.2R) + β7-specific antibody (clone FIB504)	4,5, S10
HIV + ulcerative colitis	1	PBMC	α4-specific antibody (clone 7.2R) + β7-specific antibody (clone FIB504)	8
LN HIV^+^	3	LNMC	α4-specific antibody (clone 7.2R) + β7-specific antibody (clone FIB504)	6,7
LN uninfected	3	LNMC	α4-specific antibody (clone 7.2R) + β7-specific antibody (clone FIB504)	6,7
Gut biopsy healthy control	5	LPL	α4-specific antibody (clone 7.2R) + β7-specific antibody (clone FIB504)	6, 7, S10
Gut biopsy ulcerative colitis	6	LPL	α4-specific antibody (clone 7.2R) + β7-specific antibody (clone FIB504)	6, 7, S10

PBMC, peripheral blood mononuclear cells; LNMC, lymph node mononuclear cells; LPL, lamina propria lymphocytes; VDZ, vedolizumab.

### Immune phenotypic analysis for surface markers

Cells were stained with Zombie NIR Fixable Viability stain (BioLegend, London, UK) and the following fluorochrome-conjugated surface antibodies: anti-CCR5 (clone 2D7), anti-CD27 (clone M-T271; both BD Biosciences, Heidelberg, Germany), anti-CD8 (clone RPA-T8), anti-HLA-DR (clone L243), anti-β7 (clone FIB504), anti-CD45RA (clone HI100), anti-CD127 (clone A019D5), anti-CD19 (clone H1B19), anti-CD14 (clone M5E2), anti-CCR7 (clone G043H7), anti-PD-1 (clone EH12.2H7), anti-CD4 (clone SK3), anti-CD3 (clone UCHT1), anti-CD32 (clone FUN-2), anti-CD39 (clone A1), anti-CCR9 (clone L053E8) and anti-CD69 (clone FN50; all BioLegend). The α4-specific antibody was purchased from Novus Biologicals (clone 7.2R, LLC, Centennial, CO, USA) and the α4β7-specific antibody was provided by the National Institute of Health (clone Act1, Bethesda, MD, USA). Both antibodies were unconjugated and a two-step staining with a secondary, conjugated antibody was performed (rat anti-mouse, clone X56, fluorophore BUV395, diluted 1:100, purchased from BD Biosciences, Heidelberg, Germany). The panel was compensated using single-stained Comp Beads (Anti-Mouse Ig,κ/Negative Control Compensation Particles Set, BD Biosciences). For live/dead compensation, Comp Beads stained with anti-CD14 (APC Cy-7, BioLegend) were applied. All samples were run on a BD LSR Fortessa flow cytometer with FACS Diva version 8 (BD Biosciences) on a PC.

### *In vitro* saturation and titration of α4β7 in the presence of vedolizumab

PBMC from healthy donors were thawed, washed once with PBS and counted. Cells were then transferred to RPMI supplemented with 10% FCS and Penicillin/Streptomycin (100 U/mL) and rested in an incubator overnight. The next day 1 x 10^6^ cells each were incubated with the therapeutic α4β7-specific antibody (vedolizumab, Takeda, Tokyo, Japan) at different concentrations for 90 min, washed twice and then stained with the anti-α4β7 antibody (clone Act1) or the anti-α4 antibody (clone 7.2R) in combination with a β7-specific antibody (clone FIB504).

### *In vitro* stimulation of PBMC

Cells from healthy donors were cultivated for 7 days and stimulated with either bead-bound CD3/CD28 antibodies (ThermoFisher Scientific, Waltham, USA; bead:cell ratio 1:1) or PMA/ionomycin (Sigma-Aldrich, 5 ng/mL / 500 ng/mL, respectively) + 20 U/mL IL-2 (Miltenyi, Bergisch Gladbach, Germany). To assess the effect of all-trans retinoic acid (RA) on the frequency of α4β7^+^ T cells, 100 nM RA (Enzo Life Sciences GmbH, Lörrach, Germany) in combination with bead-bound CD3/CD28 antibodies (ratio 1:1) were added to the cells. Cells were stained after 6 h, 3 days as well as after 7 days of stimulation and analysed by flow cytometry.

### Data analysis and statistics

Cytometric data were analyzed using FlowJo version 10.5.2 for Mac OS X (FlowJo, BD, Franklin Lakes, NJ, USA). Statistical analysis was performed using GraphPad Prism version 7.0c for Mac OS X (GraphPad Software, Inc., La Jolla, CA, USA). For multiple comparisons, Kruskal-Wallis and Dunn's post-test with an alpha value of 0.05 were performed. All reported p-values were multiplicity adjusted according to Dunn. To compare ranks, Wilcoxon matched-pairs signed rank tests were computed. Pearson’s correlation and Spearman’s rank correlation coefficient were applied for bivariate correlation analysis. Data are expressed as means +/- standard deviation. Frequencies in the text are described as means unless stated otherwise. A p-value of less than 0.05 was considered significant. P values were translated into asterisks as follows: *p < 0.05, **p < 0.01, ***p < 0.001, ****p < 0.0001. Not significant: ns ≥ 0.05.

## Results

### Validation of a staining protocol for T cells of healthy individuals and of patients treated with vedolizumab

**Fig 1:**
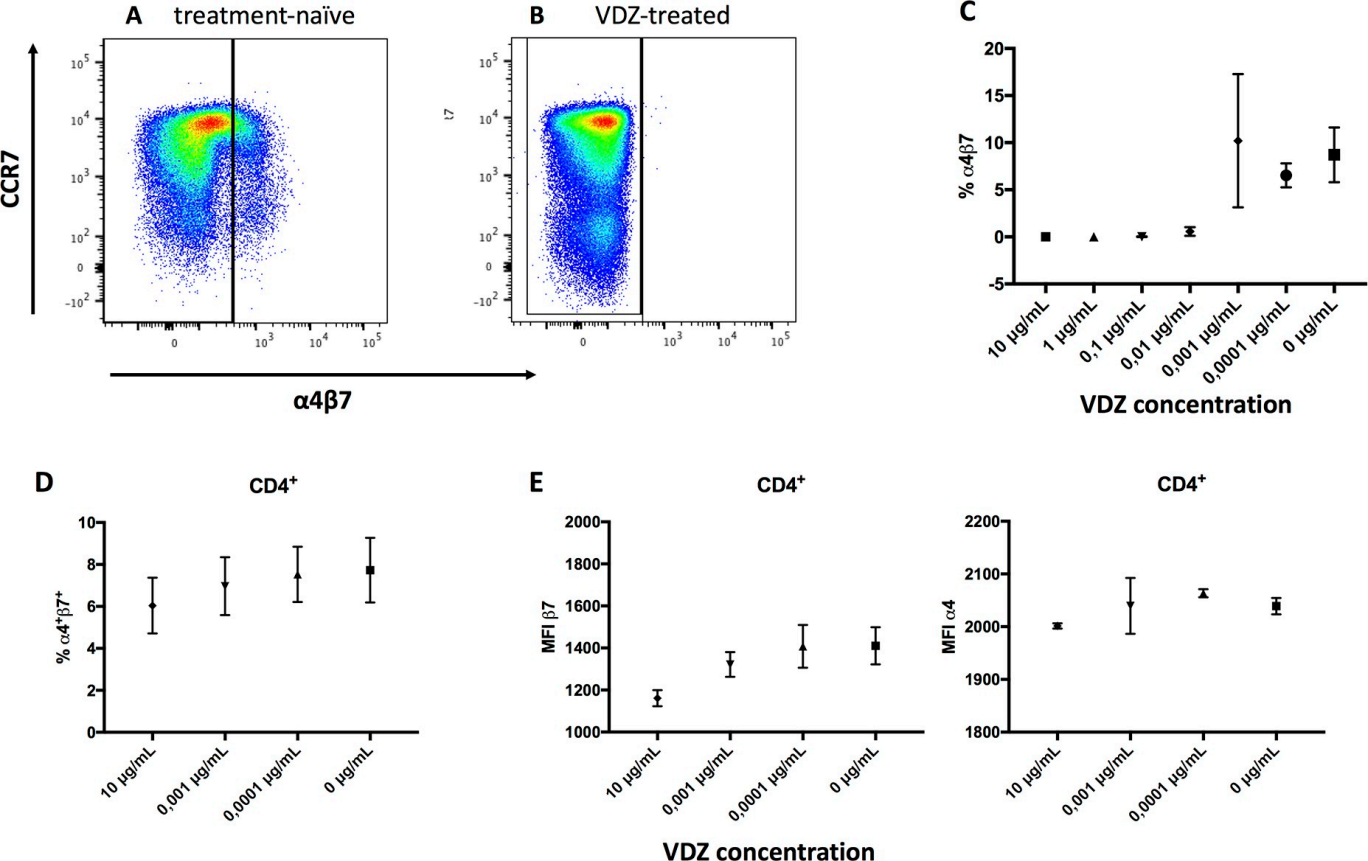
Comparison of α4β7 staining with clone Act1 vs. 7.2R + β7-specific antibody after *in vitro* incubation with vedolizumab. **A)** *Ex vivo* PBMC staining of a treatment-naïve patient stained with an α4β7-specific antibody (clone Act1). **B)** *Ex vivo* PBMC staining of a VDZ-treated patient with ulcerative colitis stained with an α4β7-specific antibody (clone Act1). **C**) Percentage of α4β7-expressing CD4^+^ T cells after i*n vitro* incubation with VDZ. Samples were incubated with the therapeutic antibody (vedolizumab) for 90 min, washed twice and then stained with the α4β7-specific antibody Act1. **D)** Percentage of α4 and β7-expressing CD4^+^ T cells upon incubation with VDZ and subsequent staining with an α4-specific antibody (clone 7.2R)+β7-specific antibody. **E)** Changes in MFI of α4 (left) and β7 (right) upon incubation with vedolizumab and subsequent staining with an α4-specific antibody (clone 7.2R)+β7-specific antibody. VDZ, vedolizumab.

For future immunological and clinical studies, it will be crucial to be able to determine the expression of α4β7 in patients treated with VDZ. Pharmacokinetic studies with vedolizumab have reported a saturation of the α4β7 integrin in peripheral blood T cells of greater than 95% between 2 and 10 mg antibody per kg bodyweight [16]. Patients receive 300 mg antibody intravenously. For a patient with a body weight of 75 kg, this means that 4 mg of antibody per kg are administered. Thus, it is assumed that practically all receptors are saturated at the dosing normally used in humans [16]. Indeed, we observed no α4β7 signal when using the common anti-α4β7 antibody, clone Act1, for staining of PBMC samples of vedolizumab-treated patients, likely due to steric hinderance (**Fig 1A and 1B**).

We conducted a series of *in vitro* experiments to determine the lowest concentration of therapeutic antibody that allowed binding of the diagnostic antibody (**Fig 1C**). Since the results of these experiments proved that it was not feasible to use the Act1 clone for the *ex vivo* analysis of patients treated with vedolizumab, we also established a staining protocol with a combination of two separate antibodies (anti-α4, clone 7.2R, anti-β7, clone FIB504). This alternative strategy led to an adequate staining signal of T cells in PBMC samples of vedolizumab-treated patients (**Fig 1D**).

To confirm that the two approaches would lead to comparable results, we stained PBMC of healthy volunteers either with the Act1 or the 7.2R clone combined with a separate β7 antibody (**S1A Fig**). Indeed, we only observed minimal differences of the measured frequencies between the two approaches and they were highly correlated (**S1B Fig).**

However, compared to previously published results (e.g. by Sivro *et al*.), there were notable differences between the staining protocols as well as the terminology [6]. The unconjugated α4β7-specific antibody (clone Act1) was combined with different secondary antibodies (PE used by Sivro *et al*. versus BUV395 used by our group) which led to a lower detectable frequency of α4β7^+^ cells in the current study. When gating against CD45RA, we mostly detected CD45RA^-^ α4β7^+^ cells, i.e. memory T cells. These cells would correspond to α4β7^high^ cells in the terminology used by e.g. Sivro *et al*. In this case, also the α4β7^high^ T-cell frequencies (between 10 and 20% reported by Sivro and colleagues) would match the total α4β7^+^ T-cell frequencies reported in the current study. In other words, using the terminology applied by Sivro *et al*., we detected the α4β7^intermediate^ CD4^+^ T cells, i.e. CD45RA^+^ α4β7^+^ cells, to a much lesser extent (see also **S2 Fig**). Since we were not able to distinguish between α4β7 ^high^ and α4β7^intermediate^ cells, we gated all α4β7^+^ cells against CCR7 in the samples from HIV patients (**S3 Fig**), or α4^+^β7^+^ double-positive cells in samples from patients with UC (**S4 Fig**) according to the respective fluorescence minus one (FMO) control.

We also conducted comparative experiments with fresh and frozen cells (**S2 and S5 Figs**). There were small differences of the frequencies between fresh and frozen cells (**S6 Fig)**, but these did not reach statistical significance.

### Frequency of α4β7^+^ CD4^+^ effector memory T cell populations differs between HIV-infected and healthy individuals

**Fig 2:**
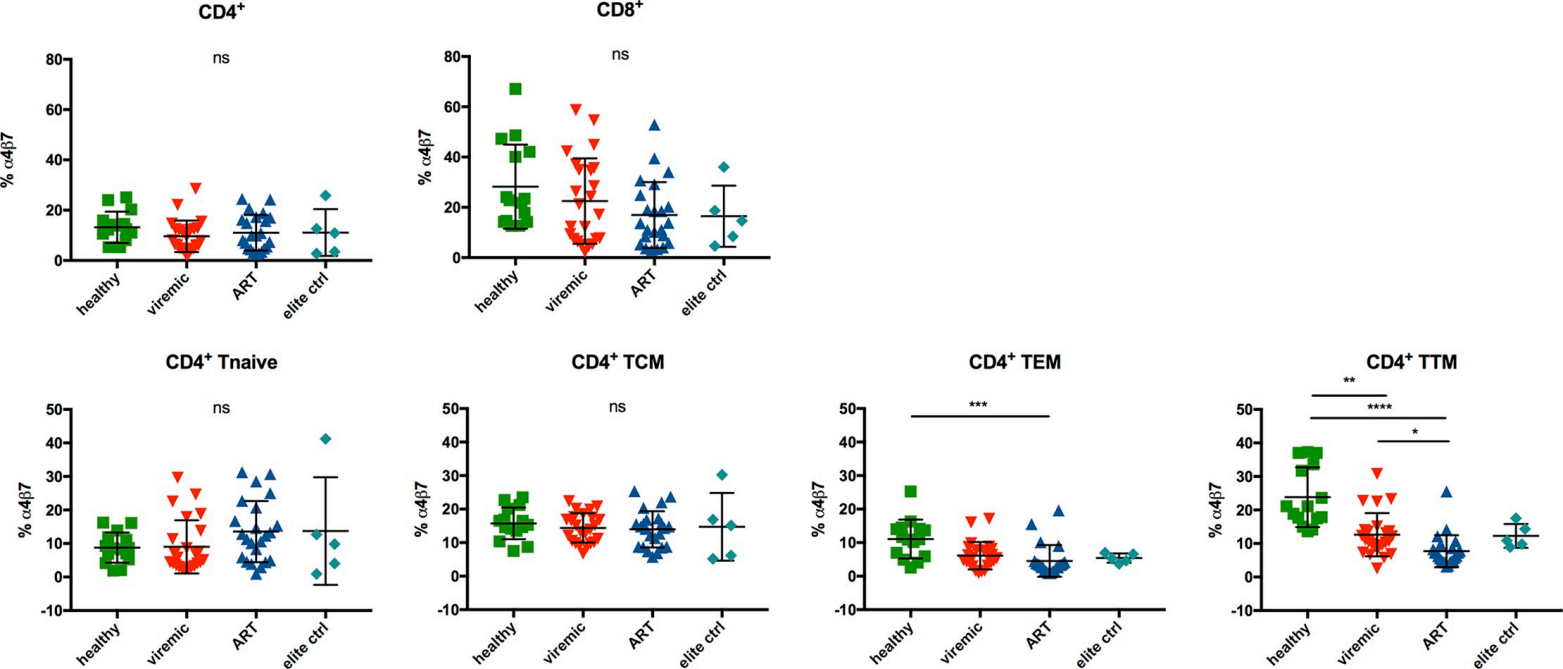
Frequency of α4β7^+^ cells differs between different effector memory CD4^+^ T-cell populations of PBMC of HIV-infected patients and healthy individuals. Frequencies of α4β7^+^ CD4^+^ T cells isolated from blood of healthy individuals (green), viremic HIV patients (red), HIV patients on antiretroviral therapy with no detectable viral load (ART, blue) and aviremic patients who did not receive ART (elite ctrl, light blue). Cryopreserved samples were thawed and directly stained with an α4β7-specific antibody (clone Act1). Data of 15 healthy controls, 24 viremic HIV patients, 23 HIV patients on ART and 5 elite controllers presented as means +/- standard deviation. ns ≥ 0.05, *p < 0.05, **p < 0.01, ***p < 0.001, ****p < 0.0001, as calculated by Kruskal-Wallis test and adjusted for multiple comparisons by Dunn’s test. Ns on top of graph indicates that none of the comparisons reached significance. ART, antiretroviral therapy; PBMC, peripheral blood mononuclear cells; TCM, central memory T cells; TEM, effector memory T cells; TTM, transitional memory T cells; ns, not significant.

Relatively little is known about the *ex vivo* expression of α4β7 on different T-cell subsets in healthy volunteers versus HIV patients with different disease course. Therefore, we compared the frequencies of α4β7^+^ CD4^+^ T cells between healthy volunteers and HIV patients (sub-stratified into viremic, ART-treated individuals and HIV elite controllers) (**Table 1 and Fig 2)**[22].

The applied gating strategy for the detection of α4β7^+^ cells in samples stained with the clone Act1 is shown in **S3 Fig**. The strategy for samples stained with an α4-specific (clone 7.2R) and a β7-specific (clone FIB504) antibody is shown in **S4 Fig.** Surprisingly, there was no significant difference of the frequency of α4β7^+^ cells within the total CD4^+^ T-cell compartment between healthy individuals, viremic and HIV patients on ART as well as HIV elite controllers (**Fig 2**).

The focus of the current study was to compare the frequency of α4β7^+^ CD4^+^ T cells, that constitute the main target cells and reservoir of HIV. However, we also analyzed CD8^+^ T cells since differences of the frequency of α4β7^+^ CD8^+^ T cells could possibly affect homing of CD8^+^ T cells to the gut. Surprisingly, we observed that the frequency of α4β7^+^ T cells was generally higher within the CD8^+^ compared to the CD4^+^ T-cell compartment (healthy: 28,23% CD8^+^ α4β7^+^ vs. 13,22% CD4^+^ α4β7^+^, p = 0,0646).

Although the percentage of total CD8^+^ T cells was significantly increased in samples of viremic and ART treated HIV patients compared to healthy controls (data not shown), the frequency of α4β7^+^ CD8^+^ T cells was lower in HIV patients on ART compared to healthy controls, but this trend did not reach statistical significance (**Fig 2**).

Next, we examined the distribution of α4β7 on CD4^+^ T cells within naïve and the different memory subsets, i.e. naïve T cells (CD45RA^+^/CCR7^+^), central memory T cells: TCM (CD45RA^-^/CCR7^+^), effector memory T cells: TEM (CD45RA^-^/CCR7^-^/CD27^-^) and transitional memory T cells: TTM (CD45RA^-^/CCR7^-^/CD27^+^). As expected, the relative frequency of naïve T cells was markedly decreased in CD4^+^ T cells of viremic HIV patients compared to healthy, ART-treated and elite controllers. In contrast, the frequency of CD4^+^ TEM of viremic HIV patients was strongly increased (**S7 Fig**).

There was no significant difference of the frequency of α4β7^+^ naïve and central memory CD4^+^ T cells between HIV patients with different clinical course (**Fig 2**). TEM as well as TTM cells of HIV patients and in particular ART-treated HIV patients expressed α4β7 at a significantly lower frequency than the respective memory subset of healthy controls with the exception of HIV elite controllers (TEM: healthy 11,08%, ART 4,53%, viremic 6,12%, elite ctrl. 5,42%; healthy vs. ART p = 0,0001—TTM: healthy 23,81%, ART 7,71%, viremic 12,61%, elite ctrl. 12,28%; healthy vs. viremic p = 0,0053, healthy vs. ART p<0,0001, ART vs. viremic p = 0,0216).

**Fig 3:**
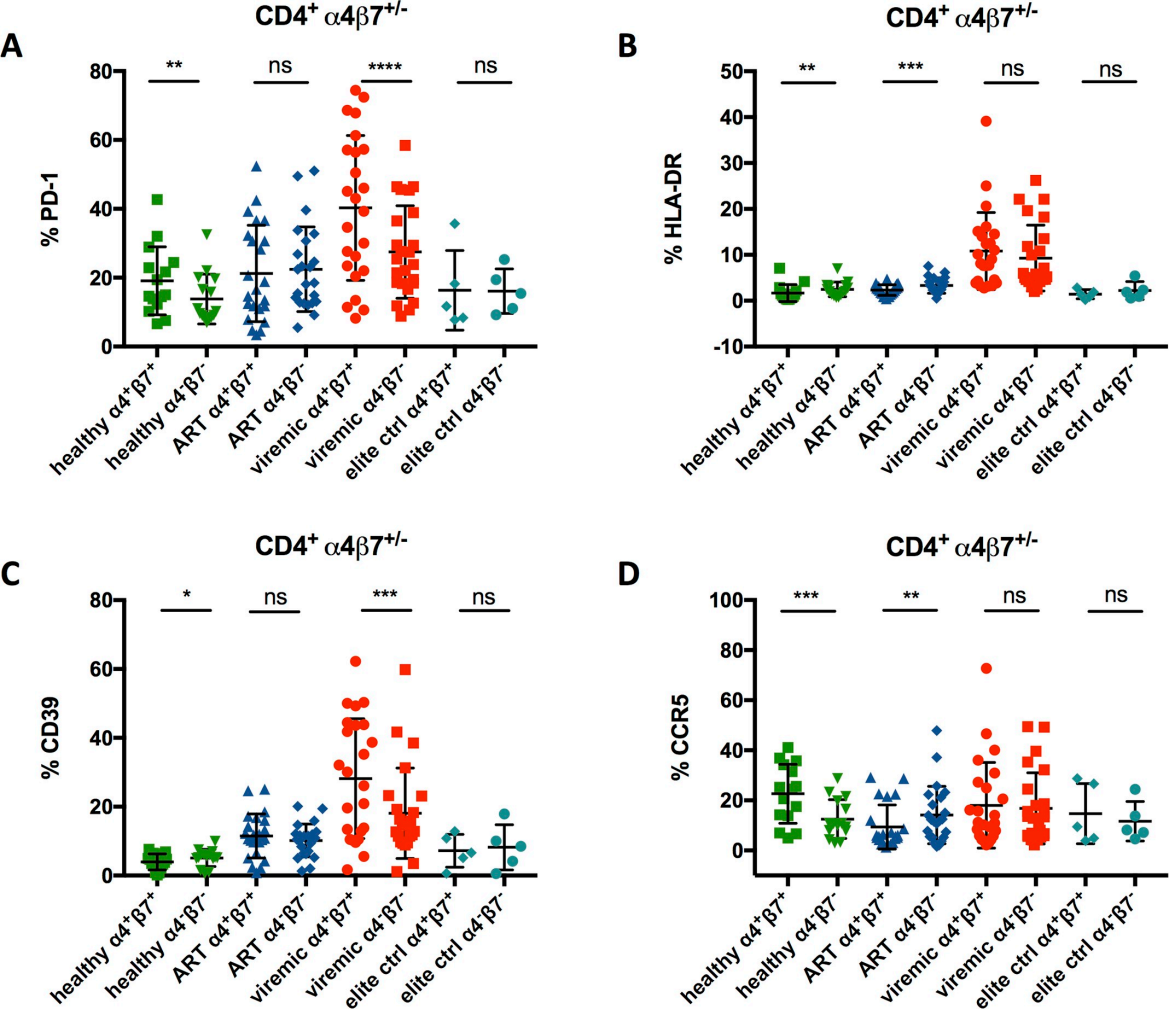
**Comparative analysis of PD-1 (A), HLA-DR (B), CD39 (C) and CCR5 (D) on α4β7**^**+**^ **and α4β7**^**-**^ **cells of healthy and HIV-infected individuals.** Frequencies of PD-1 (A), HLA-DR (B), CD39 (C) and CCR5 (D) on α4β7^+^ and α4β7^-^ CD4^+^ T cells isolated from blood of healthy individuals (green), viremic HIV patients (red), HIV patients on ART with no detectable viral load (ART, blue) and aviremic patients who did not receive ART (elite ctrl, light blue). Cryopreserved samples were thawed and directly stained with an α4β7^-^specific antibody (clone Act1). Data of 15 healthy subjects, 24 viremic HIV patients, 23 HIV patients on ART and 5 elite controllers presented as means +/- standard deviation. ns ≥ 0.05, *p < 0.05, **p < 0.01, ***p < 0.001, ****p < 0.0001, as calculated by Wilcoxon matched-pairs signed rank test. ART, antiretroviral therapy; ns, not significant.

Subsequently, we compared the characteristics of CD4^+^ α4β7^+^ versus α4β7^-^ cells in terms of activation status (HLA-DR) and exhaustion status (PD-1) as well as CD39 and CCR5 expression (**Fig 3A–3D**). Cells that express high levels of the programmed cell death protein-1 (PD-1) have been shown to be a critical source of replication-competent HI virus in patients on ART [23,24]. Thus, the molecule has been proposed to be one of the markers describing the latent reservoir and its relation to α4β7 expression on different T-cell subsets is not known. PD-1 is also a major regulator of T-cell exhaustion [25]. We observed that the frequency of PD-1-expressing cells was significantly higher in α4β7^+^ versus α4β7^-^ cells regardless of the HIV infection status (healthy: 19,11% vs. 13,8%, p = 0,0070; viremic: 40,29% vs. 27,48%, p<0,0001) (**Fig 3A)**. Representative dot plots of PD-1, HLA-DR, CD39 and CCR5 stainings are shown in **S8 Fig**.

In general, a higher frequency of HLA-DR^+^ CD8^+^ T cells of viremic HIV patients compared to healthy individuals, ART-treated patients and HIV elite controllers was detected (viremic 14,62%, healthy 3,57%, p = 0,0002; ART 3,05%, p<0,0001; elite ctrl. 2,76%, p = 0,0073; data not shown). We were also able to detect significant differences between the frequency of HLA-DR^+^ α4β7^+^ and α4β7^-^ cells (healthy: p = 0,0084; ART: p = 0,0001; **Fig 3B**).

Furthermore, we looked for differences in the frequency of the activation marker CD39 on T cells in the different HIV patient groups [26–28]. CD39 was expressed on 20–30% of peripheral CD4^+^ T cells (data not shown). Interestingly, we detected a higher CD39 frequency in α4β7^+^ CD4^+^ T cells of viremic individuals (28,18% vs. 18,09%, p = 0,0004) compared to the respective α4β7^-^ CD4^+^ T cells (**Fig 3C).**

The HIV co-receptor CCR5 was expressed at similar levels on total CD4^+^ T cells of all groups (data not shown). In healthy volunteers, we saw an elevated frequency of CCR5^+^ cells in α4β7^+^ CD4^+^ T cells (22,65% vs. 12,53%, p = 0,0009) (**Fig 3D**). In contrast, a decreased frequency of CCR5^+^ cells was measured in samples of ART-treated patients (α4β7^+^: 9,41% vs. α4β7^-^: 14,14%, p = 0,0082).

### The frequency of α4β7 on CD4^+^ T cells of HIV patients is neither correlated with viral load nor with CD4^+^ T-cell counts

It has been reported that people who highly express β7 on their CD4^+^ T cells are more susceptible to HIV infection and show faster disease progression [6]. Therefore, we investigated whether there were any correlations between CD4^+^ T-cell counts or HIV viral load and the frequency of α4β7^+^ CD4^+^ T cells. However, in this relatively small cohort we could not find a statistically significant correlation between viral load or CD4^+^ T-cell count and frequency of α4β7^+^ cells (6)(**S9 Fig**).

### The frequency of α4β7^+^ CD4^+^ T cells is decreased in the naïve CD4^+^ T-cell compartment of patients with ulcerative colitis after treatment with VDZ

**Fig 4:**
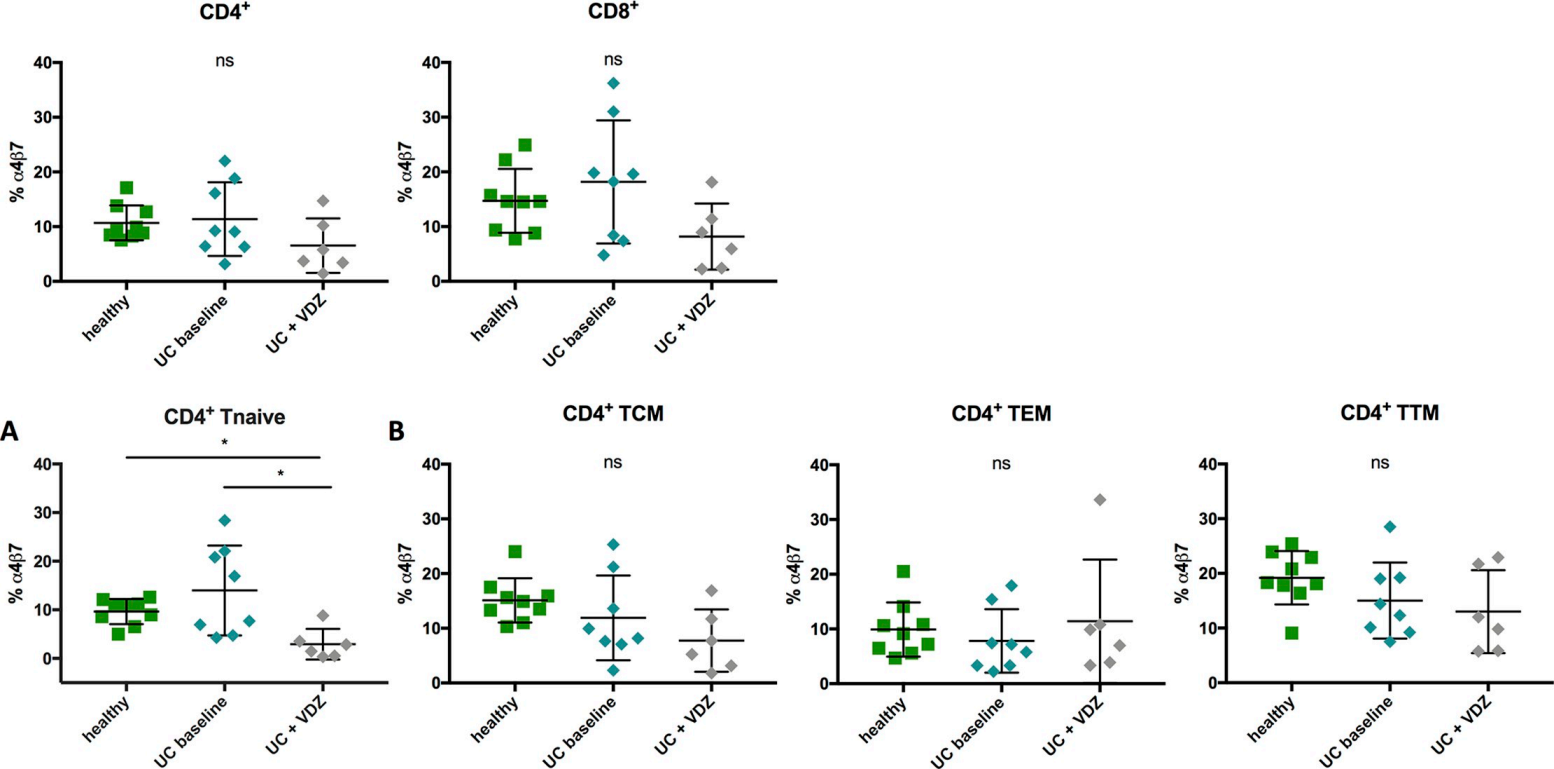
Frequency of α4β7^+^ cells is significantly decreased on naïve CD4^+^ T cells of patients with UC treated with VDZ compared to untreated UC patients. Frequencies of α4β7^+^ CD4^+^ T cells isolated from blood of healthy individuals (green), vedolizumab-naïve patients with ulcerative colitis (UC baseline, light blue) and patients with ulcerative colitis treated with vedolizumab (UC + VDZ, grey). **(A)** Frequency of α4β7^+^ CD4^+^ T naïve cells. **(B)** Frequency of α4β7^+^ within CD4^+^ memory T cells. Cryopreserved samples were thawed and directly stained with an α4-specific antibody (clone 7.2R) plus a β7-specific antibody (clone FIB504). Data of 9 healthy subjects, 8 vedolizumab- naïve patients with UC and 6 patients with UC treated with vedolizumab presented as means +/- standard deviation. ns ≥ 0.05, *p < 0.05, **p < 0.01, ***p < 0.001, ****p < 0.0001, as calculated by Kruskal-Wallis test and adjusted for multiple comparisons by Dunn’s test. Ns on top of graph indicates that none of the comparisons reached significance. VDZ, vedolizumab; UC, ulcerative colitis; TCM, central memory T cells; TEM, effector memory T cells; TTM, transitional memory T cells; ns, not significant.

In order to get a better understanding of the expression pattern of patients with inflammation of the gut and to understand the T-cell pattern in patients treated with VDZ, patients with diagnosed ulcerative colitis (UC), some of them treated with VDZ, were analysed. We studied PBMC samples obtained at different timepoints, some of them longitudinal, at baseline (before VDZ treatment initiation) and right before the administration of the follow-up infusions (week 0-2-6, followed by infusions every 8 weeks). The graphs show data at week 0 (“baseline”) and the last available timepoint of the infusions (“+VDZ”). To our surprise, there was also no significant difference of the frequency of α4β7-expressing cells of the total CD4^+^ or CD8^+^ T-cell population **(Fig 4**). However, in both compartments there was a trend towards a decreased frequency of α4β7^+^ cells in samples of VDZ-treated versus untreated patients (CD4: 6,54% vs. 11,39%, p = 0,49; CD8: 8,18% vs. 18,17%, p = 0,1592). These trends did not reach statistical significance and are in line with the results of recent studies of the T-cell pattern of IBD patients that also showed no marked changes of the number of α4β7-expressing CD4^+^ or CD8^+^ T cells of the peripheral blood [16],[29]. In contrast to HIV-infected patients, where we detected differences of the α4β7-frequency within the memory T-cell compartment between healthy and HIV infected individuals, the frequencies within the PBMC compartment of UC patients were generally comparable to samples of healthy volunteers (**Fig 4A and 4B**). However, there was a significant decrease of α4β7^+^ naïve CD4^+^ T cells between baseline samples of patients with UC and samples after treatment with VDZ (UC untreated 13,98%, UC+VDZ 2,93%, p = 0,0144) and samples of healthy controls as well as samples after treatment with VDZ (healthy: 9,65%, UC+VDZ 2,93%, p = 0,0225) **(Fig 4A)**.

**Fig 5:**
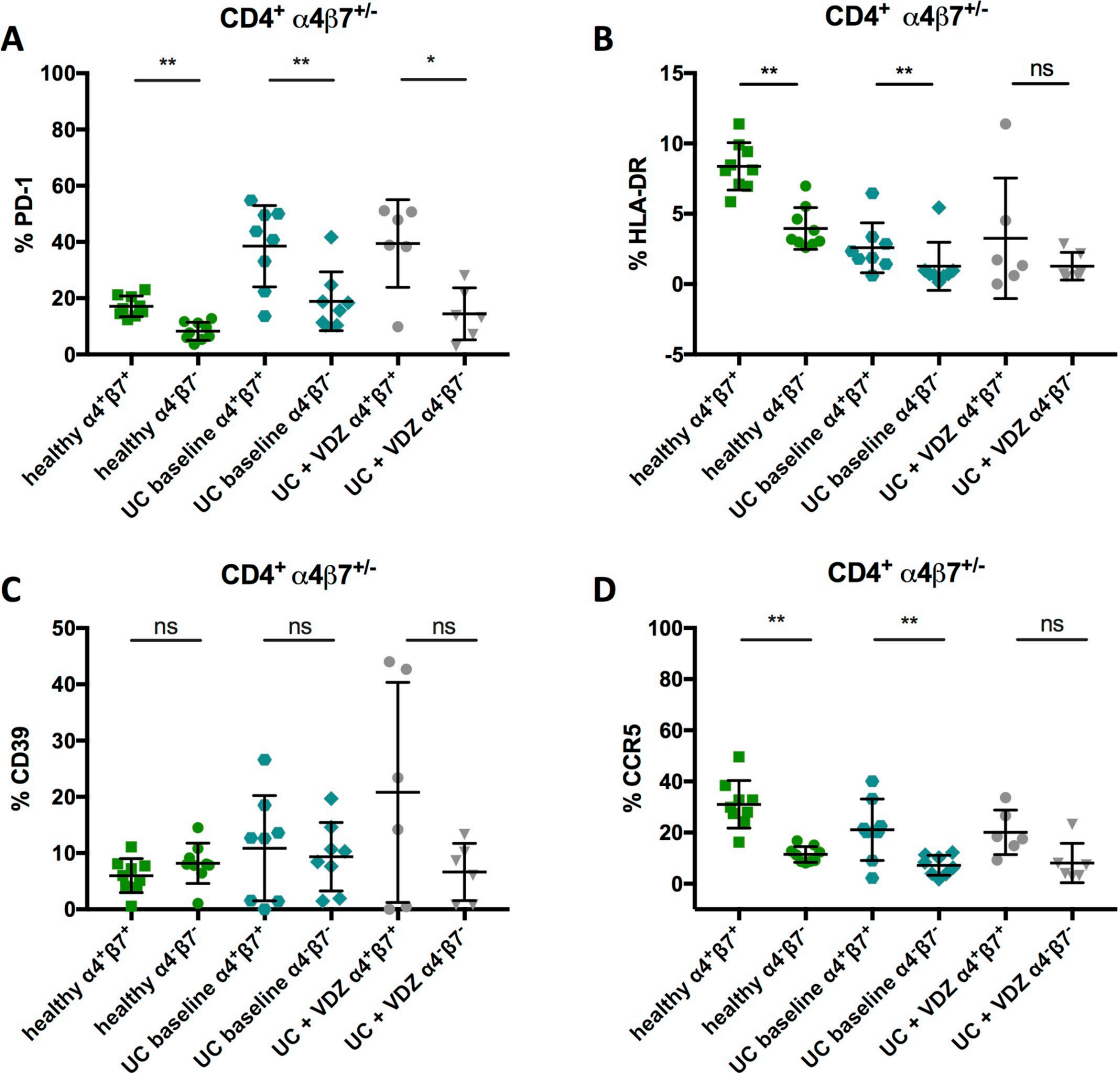
**Comparative analysis of PD-1 (A), HLA-DR (B), CD39 (C) and CCR5 (D) on α4β7**^**+**^ **and α4β7**^**-**^ **cells of healthy individuals and patients with UC.** Frequencies of PD-1 (A), HLA-DR (B), CD39 (C) and CCR5 (D) α4β7^+^ and α4β7^-^ CD4^+^ T cells isolated from blood of healthy individuals (green), vedolizumab-naïve patients with ulcerative colitis (UC baseline, light blue) and patients with ulcerative colitis treated with vedolizumab (UC + VDZ, grey) Cryopreserved samples were thawed and directly stained with an α4-specific antibody (clone 7.2R) plus a β7-specific antibody (clone FIB504). Data from 9 healthy subjects, 8 vedolizumab-naïve patients with ulcerative colitis and 6 patients with ulcerative colitis treated with vedolizumab presented as means +/- standard deviation. ns ≥ 0.05, *p < 0.05, **p < 0.01, ***p < 0.001, ****p < 0.0001, as calculated by Wilcoxon matched-pairs signed rank test. VDZ, vedolizumab; UC, ulcerative colitis; TCM, central memory T cells; TEM, effector memory T cells; TTM, transitional memory T cells; ns, not significant.

The frequency of PD-1^+^ cells was significantly higher in healthy α4β7^+^ CD4^+^ T cells (17,11% in α4β7^+^ vs. 8,27% in α4β7^-^, p = 0,0039; **Fig 5A**). The same pattern of elevated PD-1 frequency of α4β7^+^ compared to α4β7^-^ cells could be seen in untreated patients with UC (38,5% in α4β7^+^ vs. 18,91% in α4β7^-^, p = 0,0078) and after vedolizumab treatment (39,47% in α4β7^+^ vs. 14,46% in α4β7^-^, p = 0,0312). The frequency of HLA-DR^+^ CD4^+^ T cells was significantly increased in α4β7^+^ T cells of healthy individuals (8,37% in α4β7^+^ vs. 3,96% in α4β7^-^, p = 0,0039) and untreated patients with UC (2,59% in α4β7^+^ vs. 1,27% in α4β7^-^, p = 0,0078) (**Fig 5B**). There was a trend towards an increased frequency of CD39^+^ cells within α4β7^+^ compared to α4β7^-^ CD4^+^ T cells in patients with UC (both baseline and treated with VDZ) which did not reach statistical significance (**Fig 5C**). The frequency of CCR5^+^ cells of α4β7^+^ vs. α4β7^-^ CD4^+^ T cells in samples from healthy subjects and untreated UC patients was significantly higher (healthy: α4β7^+^: 31,04% vs. α4β7^-^: 11,45%, p = 0,0078; untreated: α4β7^+^: 21,12% vs. α4β7^-^: 7,23%, p = 0,0078) (**Fig 5D**).

In sum, α4β7^+^ CD4^+^ T cells of UC patients and healthy individuals showed higher frequencies of HLA-DR^+^, PD-1^+^ and CCR5^+^ cells than their α4β7^-^ counterparts. In samples of patients treated with VDZ, similar trends could be observed but these did not (with the exception of PD-1) reach statistical significance (**Fig 5A–5D)**.

### Assessment of the frequency of α4β7^+^ T cells of gut-resident CD4^+^ T-cell subsets of healthy volunteers and patients with ulcerative colitis

**Fig 6:**
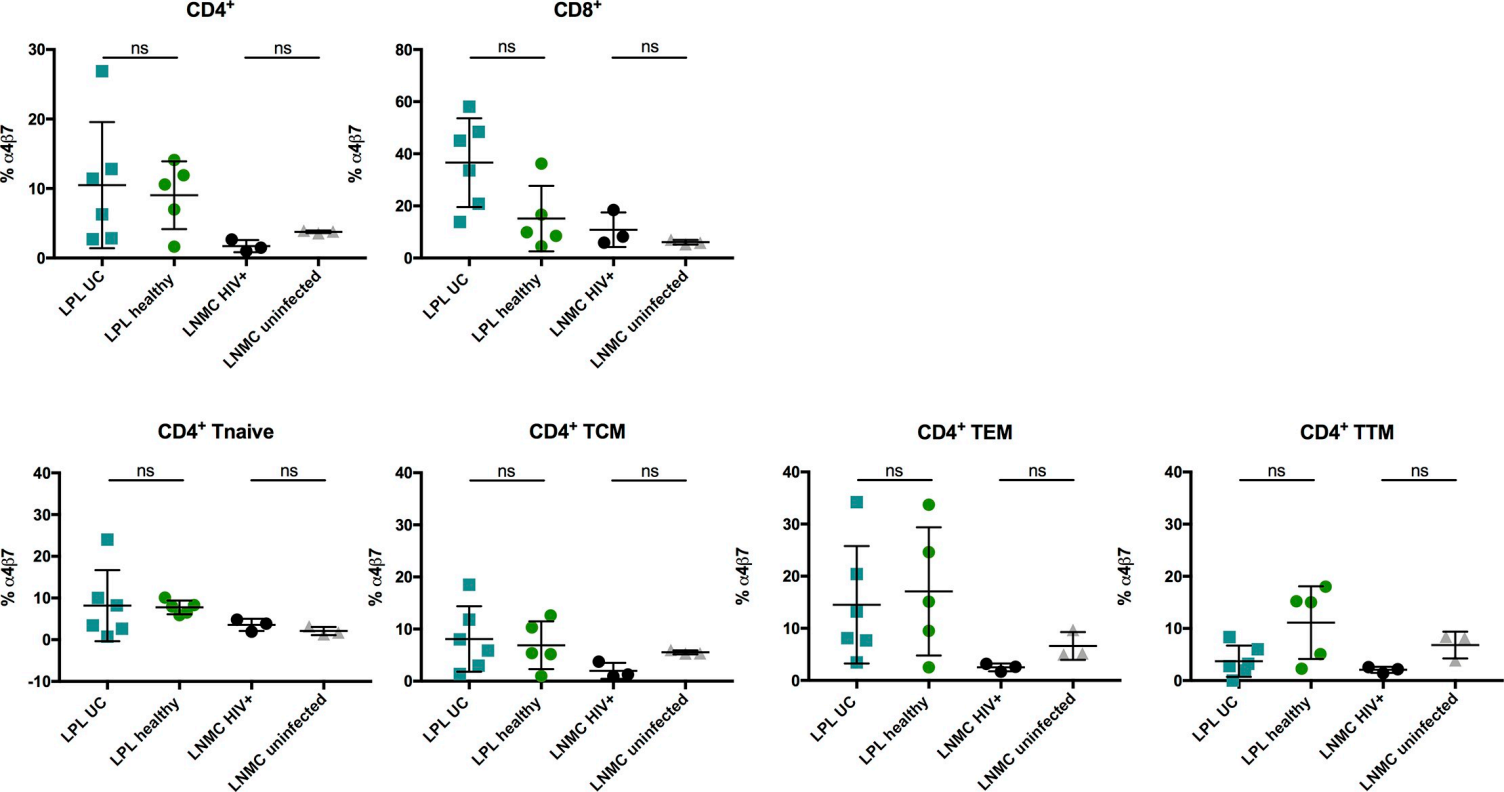
Frequency of α4β7^+^ cells does not differ between naïve and memory CD4^+^ T-cell populations of gut-derived lymphocytes of HIV-infected and healthy individuals and patients with ulcerative colitis. Frequencies of α4β7^+^ CD4^+^ T cells isolated from the sigmoid colon lamina propria of patients with ulcerative colitis (LPL UC, light blue), healthy individuals (LPL healthy, green) as well as α4β7 frequencies of mononuclear cells isolated from lymph nodes of HIV positive (LNMC HIV+, black) and HIV negative (LNMC uninfected, grey) individuals. LPL were isolated from gut biopsies overnight and stained freshly, whereas LNMC were cryopreserved. Both sample types were stained with an α4-specific antibody (clone 7.2R) plus a β7-specific antibody (clone FIB504). Data of 6 patients with ulcerative colitis, 5 healthy individuals as well as 3 HIV positive patients and 3 HIV negative individuals presented as means +/- standard deviation. ns ≥ 0.05, *p < 0.05, **p < 0.01, ***p < 0.001, ****p < 0.0001, as calculated by Mann-Whitney test. LPL, lamina propria lymphocytes; LNMC, lymph node mononuclear cells; UC, ulcerative colitis; TCM, central memory T cells; TEM, effector memory T cells; TTM, transitional memory T cells; ns, not significant.

A recent report has demonstrated an attenuation of lymphoid aggregates in the terminal ileum after VDZ therapy [13]. We conducted preliminary additional experiments with readily available tissue samples and examined mononuclear cells of the sigmoid colon (LPL) of 6 patients with UC and 5 healthy individuals as well as LNMC of 3 HIV-positive and 3 HIV-negative individuals (**Fig 6)**.

The frequencies of α4β7^+^ LPL CD4^+^ T cells were lower in samples of healthy individuals compared to those of UC patients (healthy: 9,05% vs. UC: 10,49%). In samples of patients with UC, the frequencies of α4β7^+^ CD4^+^ T cells were lower compared to the levels measured in PBMC (healthy: 10,69%, UC: 11,39%, **Fig 4**). The frequency of α4β7^+^ CD8^+^ T cells was generally higher than that of the corresponding CD4^+^ T-cell compartment (LPL UC 36,62%, LPL healthy 15,14%, LNMC HIV 10,85%, LNMC uninfected 6,07%) (**Fig 6)**.

The frequency of α4β7^+^ cells within different LPL CD4^+^ T cell subsets was similar between samples of patients with UC and healthy individuals, whereas the highest frequency could be detected in the CD4^+^ TEM subset regardless of the disease status. We found comparable frequencies of α4β7^+^ in naïve T cells (LPL UC: 8,17%, LPL healthy: 7,75%), TCM (LPL UC: 8,09%, LPL healthy: 6,89%), TEM (LPL UC: 14,51%, LPL healthy: 17,08%) and TTM (LPL UC: 3,72%, LPL healthy: 11,11%).

### Assessment of the frequency of α4β7^+^ T cells of lymph nodal CD4^+^ T-cell subsets of HIV patients compared to uninfected controls

In another set of experiments, we measured the frequency of α4β7^+^ CD4^+^ T cells of LNMC of HIV-infected vs. uninfected individuals. Only 3,77% of the CD4^+^ LNMC of HIV-negative controls were α4β7^+^ (HIV infected: 1,71%). The frequency of α4β7^+^ cells was generally lower on LNMC than on LPL for all studied subsets regardless of the infection status **(Fig 6)**. In uninfected LNMC, we measured a frequency of α4β7^+^ cells of 2,09% in naïve CD4^+^ T cells, 5,54% in TCM, 6,61% in TEM and 6,83% in TTM (HIV: 3,54% α4β7 in naïve CD4^+^ T cells, 1,99% in TCM, 2,5% in TEM and 2,07% in TTM).

### Ratio of CCR9 and α4β7 is inverted on peripheral versus gut-resident CD4^+^ T cells of healthy individuals and patients with UC

We did not see a major difference between the frequency of peripheral versus gut-resident α4β7^+^ T cells. One potential reason for this finding is that α4β7 is described to be downregulated on T cells after migration to the GALT [30]. As a second homing marker, we analyzed the frequency of CCR9, a chemokine receptor, which also facilitates migration to the GALT via CCL25 binding, on peripheral versus gut-resident CD4^+^ T cell subsets **(S10 Fig)** [31].

The frequencies of CCR9^+^ CD4^+^ T cells were generally higher in LPL than on peripheral blood cells and the frequencies of CCR9^+^ LPL tended to be higher than of α4β7^+^ LPL.

Interestingly, the frequencies of CCR9^+^ in comparison to α4β7^+^ CD4^+^ T cells were inversed in peripheral blood and the gut. In PBMC (**S10A Fig**), there was a higher frequency of α4β7^+^ CD4^+^ T cells in healthy individuals (p = 0,0039) and patients with UC who were treatment-naïve (p = 0,0078). In UC patients treated with VDZ, the difference between the frequency of CCR9^+^ and α4β7^+^ cells was smaller and non-significant (p = 0,6875). In LPL of patients with UC, the frequency of CCR9^+^ CD4^+^ T cells was significantly higher than the frequency of α4β7^+^ CD4^+^ T cells (p = 0,0312) **(S10B Fig)**.

Again, the situation was different for LNMC of HIV-positive and HIV-negative individuals, where the frequencies of CCR9^+^ and α4β7^+^ cells were similar and showed an overall lower level.

### Frequency of CCR5^+^, CD39^+^, HLA-DR^+^ and PD-1^+^ gut-resident CD4^+^ T cells of patients with ulcerative colitis and healthy individuals

**Fig 7:**
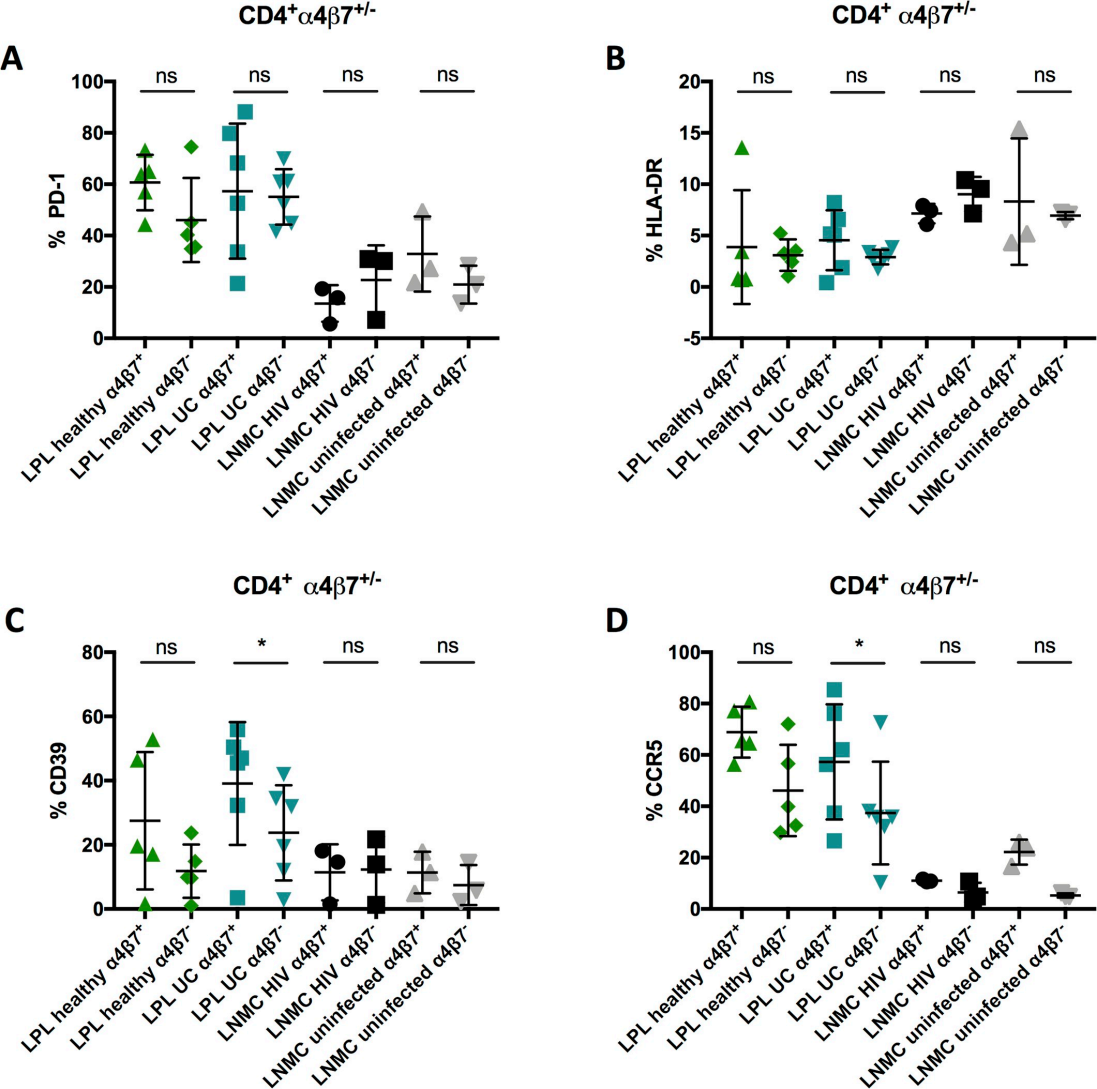
Comparative analysis of PD-1, HLA-DR, CD39 and CCR5 of α4β7^+^ and α4β7^-^ LPL and LNMC of healthy individuals and patients with UC. Frequencies of PD-1 (A), HLA-DR (B), CD39 (C) and CCR5 (D) α4β7^+^ and α4β7^-^ CD4^+^ T cells isolated from the sigmoid colon lamina propria of patients with ulcerative colitis (LPL UC, light blue), healthy individuals (LPL healthy, green) as well as α4β7 frequencies of mononuclear cells isolated from lymph nodes of HIV positive (LNMC HIV+, black) and HIV negative (LNMC uninfected, grey) individuals. LPL were isolated from gut biopsies overnight and stained freshly, whereas LNMC were cryopreserved. Both sample types were stained with an α4-specific antibody (clone 7.2R) plus a β7-specific antibody (clone FIB504). Data of 6 patients with ulcerative colitis, 5 healthy individuals as well as 3 HIV positive patients and 3 HIV negative individuals presented as means +/- standard deviation. ns ≥ 0.05, *p < 0.05, **p < 0.01, ***p < 0.001, ****p < 0.0001, as calculated by Wilcoxon matched-pairs signed rank test. LPL, lamina propria lymphocytes; LNMC, lymph node mononuclear cells; UC, ulcerative colitis; TCM, central memory T cells; TEM, effector memory T cells; TTM, transitional memory T cells; ns, not significant.

In analogy to the observations made in the case of peripheral CD4^+^ T cells, α4β7^+^ LPL of patients with UC and healthy individuals tended to have higher frequencies of CCR5^+^, CD39^+^ and PD-1^+^ cells (**Fig 7A–7D**). These differences only reached significance between the frequencies of CD39^+^ and CCR5^+^ α4β7^+^ and α4β7^-^ LPL of patients with UC (p = 0,0312 for both samples).

### The frequency of α4β7^+^ CD4^+^ T cells of healthy volunteers increases upon *in vitro* stimulation

To determine whether the frequency of α4β7^+^ T cells is directly correlated with the activation status of the T cells, we stimulated PBMC of healthy donors with bead-bound CD3/CD28 or PMA/ionomycin *in vitro* for up to 7 days (**S11 Fig**).

After 7 days of stimulation with bead-bound CD3/CD28, the frequency of CD4^+^ T cells was similar compared to unstimulated samples (**S11A Fig**), whereas the frequency of α4β7^+^ CD4^+^ T cells was significantly increased (66,37% vs. 5,59%, p = 0,0286; **S11B Fig**). After 6 hours of stimulation (day 0) and at day 3, no significant differences between the groups could be observed. The density of α4β7 on CD4^+^ T cells as measured by the mean fluorescence intensity (MFI) was decreased on day 3 in samples stimulated with PMA and ionomycin compared to unstimulated controls (p = 0,0286) (**S11C Fig)** and increased significantly after 7 days of stimulation (p = 0,0286).

As an additional control we examined samples that were stimulated with 100 nM all-trans retinoic acid (RA) which can be produced by gut-associated dendritic cells and has been described as a modulator of α4β7 expression in lymph nodes [32,33]. After 7 days of stimulation, the frequency of α4β7^+^ CD4^+^ T cells was significantly higher compared to unstimulated samples (89% vs. 5,59%, p = 0,0286; **S11D Fig**).

In sum, we observed an activation- and retinoic acid-dependent upregulation of the α4β7^+^ CD4^+^ T cell frequency *in vitro* in PBMC stimulated with bead-bound CD3/CD28 as well as retinoic acid. Furthermore, the expression level of α4β7 increased upon stimulation with PMA/ionomycin and RA (data not shown).

Interestingly, although HLA-DR^+^ cells seemed to be more frequent among α4β7^+^ CD4^+^ T cells than among α4β7^-^ ones (**Fig 5B**), there was no statistically significant correlation between general activation of the immune system (as indicated by the frequency of HLA-DR^+^ CD8^+^ T cells) and frequency of α4β7^+^ cells in viremic and patients on ART. Also, looking at α4β7^+^ T cells, there was no correlation between the frequency of HLA-DR^+^ and α4β7^+^ cells and HIV viremia (**S9A Fig**). There were too few data points to correlate T cell activation and α4β7 frequency in samples from UC patients.

### Longitudinal study of α4β7 expression on CD4^+^ T cells of an HIV-positive patient with ulcerative colitis treated with vedolizumab

**Fig 8:**
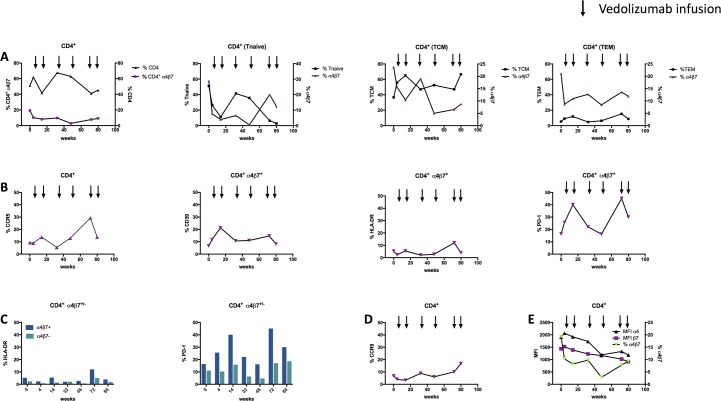
Longitudinal study of the frequencies of α4β7^+^ CD4^+^ and CD8^+^ T cells from a HIV patient with concomitant ulcerative colitis. Analysis of PBMC of a HIV-positive patient who was diagnosed with UC after being on ART for 17 years with well-controlled HIV viremia. Blood samples were taken before and during treatment with vedolizumab (at week 4, 14, 32, 48, 72 and 80 after treatment initiation). (A) Frequencies of α4β7^+^ CD4^+^ T cells within different CD4^+^ T cell compartments over the course of therapy. (B) Frequencies of expression of activation and exhaustion markers as well as the HIV co-receptor CCR5 during the VDZ therapy. (C) Frequencies of HLA-DR^+^ and PD-1^+^ α4β7^+^ versus α4β7^-^ CD4^+^ T cells. (D) Course of the frequency of CCR9^+^ CD4^+^ gut-homing T cells. (E) Expression levels of α4 and β7 on CD4^+^ T cells as indicated by mean fluorescence intensity (MFI) during VDZ therapy in comparison to the frequency of α4β7^+^ CD4^+^ T cells. Cryopreserved samples were thawed and directly stained with an α4-specific antibody (clone 7.2R) plus a β7-specific antibody (clone FIB504). Arrows indicate the timepoints of vedolizumab infusions. PBMC, peripheral blood mononuclear cells; TCM, central memory T cells; TEM, effector memory T cells; TTM, transitional memory T cells; ns, not significant; VDZ, vedolizumab; MFI, mean fluorescence intensity.

We collected PBMC of an HIV-positive patient who was diagnosed with UC after being on ART for 17 years, with well-controlled HIV viremia (current regimen: Tenofovir Alafenamide/Emtricitabine and Dolutegravir). We analysed samples before and during treatment with vedolizumab (at week 4, 14, 32, 48, 72 and 80 after treatment initiation) (**Fig 8A–8E**). The viral load remained below level of detection and CD4^+^ counts ranged from 749–1239 cells/μL throughout the study. The colitis which was described to be of intermediate activity was first being treated with cortisone and Mesalazine which were both discontinued before the start of the vedolizumab therapy. Vedolizumab was administered safely and without any serious adverse events and the patient reported an amelioration of symptoms during therapy which could be related both to clinical parameters (e.g. CRP values) and macroscopic and microscopic findings of the endoscopic examination.

We detected a stable decrease of the frequency of both CD4^+^ and CD8^+^ α4β7^+^ T cells over time. (**Fig 8A,** CD8 data not shown). The frequency of α4β7^+^ cells was generally higher on CD8+ T cells (mean of all timepoints 16,53% of CD8^+^ vs. 9,63% of CD4^+^). The frequency of total CD4^+^ T cells declined at week 14 (from 61,8% to 41,7%) and again at week 72 (from 62,8% to 41%).

Also, at week 72, the general immune activation (percent of HLA-DR^+^ CD8^+^ T cells) rose from 1,45% to 8,2%. Furthermore, levels of PD-1 and HLA-DR on α4β7^+^ cells peaked (PD-1 from 25,6% to 40% at week 14 and from 16,2% to 45,1% at week 72; HLA-DR from 2,33% to 5,42% at week 14 and from 2,74% to 12% at week 72) (**Fig 8B**). Interestingly, the frequency of CCR9^+^ CD4^+^ T cells increased continuously during therapy (from 4,02% at week 4 to 16,7% at week 80) (**Fig 8D**).

Similar to what could be observed in the patients with UC who are HIV-negative, the frequency of α4β7^+^ naïve CD4^+^ T cells decreased under VDZ therapy (**Figs 4A and 8A**), whereas the frequency of naïve T cells recovered after week 14. However, at weeks 72 and 80, we measured a drastic decrease of naïve CD4^+^ T cells (from 35,7% at week 48 to 6,34% at week 72 and 2,78% at week 80). Concurrently, the frequency of α4β7^+^ naïve CD4^+^ T cells ascended from 0,45% to 20% and 12,3% (week 80). The frequency of CD4^+^ TCM remained fairly stable during treatment. Except one peak at week 32, where the frequency rose from 10,4% to 19% for unknown reasons, the frequency of α4β7^+^ cells steadily decreased (**Fig 8A**). The frequency of TEM peaked at weeks 14 and 72 (11,8% and 15,2%). This did not interfere with the frequency of α4β7^+^ TEM cells, that showed a steady decrease over time could be observed (similar to the development of the frequency of the α4β7^+^ TCM subset).

## Discussion

The integrin α4β7 is a heterodimer that regulates intestinal T-cell homing via binding of MAdCAM-1 on endothelial venules in the GIT. Originally mainly of interest as a target for the treatment of inflammatory bowel diseases, α4β7 and its modulation via integrin-specific antibodies such as VDZ have gained increased attention by researchers within the HIV field [6,9–11,13,29,34].

In the current study we designed flow cytometric panels to specifically examine the frequency of α4β7^+^ T cells of healthy volunteers, patients diagnosed with HIV infection at different stages including elite controllers on one hand and patients with ulcerative colitis, some of them treated with vedolizumab, on the other. Furthermore, we studied differentiation, exhaustion as well as activation status of these T cells. In contrast to the commonly used clone Act1, the combination of an α4- and a β7-specific antibody permitted the detection of α4β7 surface expression on T cells of patients who were treated with vedolizumab. In line with previously published data, we only find minor differences of the frequency of α4β7 on total CD4^+^ T cells regardless of differentiation, disease status (either HIV or UC) or treatment with VDZ.

Nevertheless, we observed minor differences between the expression pattern of certain CD4^+^ T-cell subsets: namely we detected decreased frequencies of α4β7^+^ cells within the memory compartment (TEM and TTM CD4^+^ T cells) of HIV patients.

These data fit well with a recent study reporting an increased susceptibility of α4β7^+^ memory CD4^+^ T cells as early targets of HIV infection [35]. Indeed, effector CD4^+^ T cells are more likely to be infected and depleted due to their activation status [35],[36].

Interestingly, the decreased frequency of α4β7^+^ CD4^+^ T cells was more pronounced in patients on ART than in viremic patients and this low frequency did not recover during therapy. A study by Sivro *et al*. reported that ART initiated as early as in Fiebig stage I or II failed to restore β7^high^ cells in PBMC during two years of treatment [6].

We also detected differences regarding the expression of the HIV co-receptor CCR5, that is expressed at a higher frequency on α4β7^+^ cells of healthy individuals which could offer another explanation for the higher infectibility of these CD4^+^ T cells [37–39]. A similar trend could be observed in LPL samples. In LPL, the frequency of CCR5^+^ T cells was elevated within α4β7^+^ CD4^+^ T cells of healthy individuals and patients with UC. Strikingly, in healthy volunteers, the frequency of CCR5^+^ α4β7^+^ CD4^+^ LPL was about 3,5 times higher than the frequency of peripheral CCR5^+^ α4β7^+^ CD4^+^ T cells. It has been reported that CD4^+^ T cells upregulate CCR5 in response to MAdCAM-1 co-stimulation [40], so it is intriguing to speculate whether the higher frequency of CCR5^+^ α4β7^+^ CD4^+^ T cells in the gut could be a result of the interaction of α4β7 and MAdCAM-1 during diapedesis.

It has been reported that activation correlates with the frequency of α4β7^+^ cells [6]. Although this can be reproduced *in vitro* upon stimulation with bead-coupled anti-CD3 and anti-CD28, we were not able to detect any correlation in viremic or patients on ART in this study. However, this could also be due to the lacking statistical power to see this effect in this rather small cohort.

In the second part of the current study, we examined possible changes of the T-cell compartment after initiation of a VDZ therapy in HIV-negative patients diagnosed with UC. We observed differences of the frequency of α4β7^+^ cells between CD8^+^ T cells and between naïve CD4^+^ T cells of VDZ-treated versus untreated patients with UC. In particular, the frequency of α4β7^+^ cells was elevated on naïve CD4^+^ T cells of untreated patients and then dropped under VDZ therapy (**Fig 4A**). The changes we detected in terms of the frequency of α4β7^+^ naïve CD4^+^ T cells of UC patients are in line with published data [41]. Cimbro *et al*. could show that α4β7 on naïve T cells was induced and activated by IL-7 [42]. After activation, the cells differentiate into memory phenotypes. So, it is likely that naïve cells are more sensitive to modifications of the integrin than (effector) memory cells, where we did not detect major changes. This could not only possibly predict the longer time of remission of active UC after VDZ mono-therapy initiation [43] since gut-resident memory T cells would not be immediately affected, but could also pose a problem for future HIV cure strategies using VDZ since mainly naïve CD4^+^ T cells seem to decrease during therapy which are not classical targets of the HI virus [44–46].

Interestingly, the frequency of HLA-DR^+^ cells of the α4β7^+^ CD4^+^ T-cell subset was lower in untreated UC patients compared to healthy controls. Also, the frequency of α4β7^+^ LPL of UC patients was neither elevated compared to α4β7^+^ CD4^+^ LPL of healthy volunteers nor elevated compared to the equivalent population in PBMC (UC baseline). This indicates that the activation of LPL and PBMC is not necessarily directly linked to the general higher level of immune inflammation. As expected, we observed overall lower frequencies of α4β7^+^ cells within LNMC compared to LPL. The migration to peripheral lymph nodes is mediated by the integrin αLβ2 and intercellular adhesion molecule-1 (ICAM-1) [30]. α4β7 has been described to be upregulated by all-trans-retinoic acid exclusively in mesenteric lymph nodes [32]. During priming in mesenteric lymph nodes and Peyer’s patches, retinoic acid converted by dendritic cells induces the expression of CCR9 and α4β7 on naïve B and T cells [32,33].

We observed inverted ratios of CCR9 and α4β7 in PBMC and gut. The diminished frequency of α4β7^+^ cells in the GALT is in line with studies describing the downregulation on T cells after migration to the GALT [30]. CCR9 has been investigated as potential drug target to hinder the migration of leukocytes, unfortunately with poor outcome [47]. However, it mainly facilitates the migration to the small intestine and has been hypothesized to play a limited role during inflammation since it might be a predominantly homeostatic molecule [48]. Yet, another study by Trivedi and colleagues has shown that the colonic expression of the CCR9 ligand CCL25 is upregulated in patients with active colitis and also correlates with the endoscopic Mayo score [49]. Hence, a combinational therapy targeting both α4β7^+^ and CCR9^+^ T cells might still be feasible.

Finally, we described the distribution of α4β7 on different T-cell subsets during VDZ therapy in samples from a HIV positive patient with well controlled viremia under ART who was recently diagnosed with UC. This patient safely tolerated therapy and there was a steady decrease of α4β7^+^ naïve T cells which was accompanied by a decrease in symptoms until week 72 of follow-up. The kinetics of α4β7 and CCR9 frequencies of CD4^+^ T cells during VDZ therapy was similar, indicating that also other gut-homing cells can be affected by VDZ or that both molecules are co-expressed.

Towards the end of the follow-up period, there was a sharp decline in the frequency of CD4^+^ naïve T cells which was accompanied by an increase of α4β7^+^ naïve T cells and a peak of HLA-DR^+^ CD8^+^ T cells. Since a rebound in viremia was excluded it could be that the patient has started to reject the therapeutic antibody. Indeed, it is assumed that the patient has produced antibodies against VDZ, as serum levels of the therapeutic antibody were measured at strikingly subtherapeutic levels (data not shown) [50].

It should be noted that samples of HIV-infected patients (viremic, ART, elite controllers) as well as the healthy controls that are depicted in the respective graphs have been stained with the α4β7-specific clone Act1. Samples of patients with UC have been stained with two separate antibodies because samples from VDZ-treated patients cannot be analyzed with the Act1 antibody (see Table 2). Also, a recent study by Perciani *et al*. has also demonstrated that it is incorrect to assume that all β7^hi^ populations isolated from mucosal tissue are α4β7^hi^ as well [17].

We have assessed the comparability of the two staining panels and have found a good correlation between the two staining approaches (**S1 Fig**). Thus, comparisons within the groups (HIV versus matching healthy controls and UC versus matching healthy controls) can be made.

However, the frequencies of total CD4^+^ α4β7^+^ T cells reported in this work are considerably lower (10 to 25%) than results obtained by other groups, e.g. Kelley *et al*. who found frequencies between 15 and 40% in peripheral blood [6,51] and the α4β7^+^ T cells gated in the current study largely seem to overlap with the α4β7^high^ T-cell populations defined by other groups [6,51] (see also **Figs 2 and 4**).

Another limitation of the current study is that the group of analyzed patients with UC was relatively small, fairly heterogenous (2 patients were diagnosed with PSC and one was a liver transplant patient) and that no standardized clinical staging (i.e. MAYO score) was available for all patients and timepoints and gut biopsies were only obtained from the sigmoid colon and not from other parts of the colon. Some received concomitant medication for the IBD or other diseases (see **Table 1**). Finally, we were not able to obtain matched PBMC and gut samples. Given these pronounced limitations and small comparative cohort of UC patients, it was surprising to still detect consistent changes of T-cell patterns under VDZ treatment. Lastly, due to the limitation of markers that could be stained in one flow cytometry panel, we did not have the chance to define frequencies of α4β7^+^ Tregs and α4β7^+^ Th17 T-cell subpopulations in PBMC and LPL of patients treated with VDZ, or surface expression of α4β7 on other lymphocyte populations in general [52,53].

The results of our and previous studies have major implications since they suggest that the therapeutic effect of VDZ in UC might also be caused by modulation of innate immune cells rather than the T-cell compartment that overall only shows small shifts of frequency and phenotype [29,54,55]. The immunological changes caused by VDZ may be more a generally concerted “anti-inflammatory phenotype” of different cell types in the gut.

Of interest, results of different studies suggest that there might be an α4β7/MAdCAM-1-independent way of trafficking of pathogenic CD4^+^ T cells: although viral load could be well controlled by rhesus monkeys treated with a similar therapeutic antibody and then infected with SIV, the number of α4β7 cells in the gut only mildly decreased [11]. Also, antibody-mediated blockade of the β7 integrin, the α4β7 heterodimer, MAdCAM-1 or L-selectin did not dampen inflammation in a set of mouse experiments with chronic ileitis [55].

In the current study we established flow cytometric staining panels for the analysis of the α4β7 integrin in patients who received treatment with VDZ. This allowed us to analyze different blood and tissue samples in diverse patient groups in order to get a better understanding of the general expression pattern and changes to be expected under vedolizumab therapy.

In follow-up studies of patients with chronic IBD or HIV respectively, additional T-cell subpopulations (Tregs, Th17 cells, γδ T cells and MAIT cells), B cells, as well as NK cells, dendritic cells and monocytes have to be analyzed regarding their α4β7 expression in PBMC and the gut. Also, large prospective studies of paired PBMC and LPL samples from UC patients treated with different regimens comparing clinical outcome and immunological signature have to be performed [43].

In terms of HIV infection, next logical steps will be the determination of the viral reservoir of CD4^+^ T cells stratified by α4β7 expression in HIV patients treated with VDZ [13,56].We also plan to conduct HIV-specific T cell staining via MHC class I+II tetramers in order to check for possible correlations between integrin-expression and HIV-specific T-cell responses. Finally, immunohistochemistry of gut biopsies in order to determine exact numbers of infiltrating cells will be conducted.

The results of the current study will serve as an important data set for the design of future immunomonitoring panels in clinical trials which e.g. evaluate the use of vedolizumab in HIV infection. Our data confirm previous studies suggesting that mechanisms other than blockade of the α4β7/MAdCAM-1 interaction of T cells might be responsible for the amelioration of symptoms in patients with IBD and the beneficial effects of vedolizumab in respect to viral control seen in rhesus macaques infected with SIV.

## Supporting information

S1 Table Cohort statistics of patients with ulcerative colitis. (PDF)

S1 Fig Comparison of the α4β7-specific clone Act1 and the α4-specific clone 7.2R in combination with a separate β7-specific antibody. (PDF)

S2 Fig Comparison of fresh and frozen cells stained with the α4β7-specific antibody (clone Act1). (PDF)

S3 Fig Gating strategy for CD4^+^ α4β7^+^ T cells stained with the α4β7-specific (clone Act1) antibody. (PDF)

S4 Fig Gating strategy for CD4^+^ α4β7^+^ T cells stained with an α4-specific (clone 7.2R) and a β7-specific (clone FIB504) antibody. (PDF)

S5 Fig Comparison of fresh and frozen cells stained with the α4-specific clone 7.2R in combination with β7-specific antibody (FIB504). (PDF)

S6 Fig Comparison of fresh and frozen cells stained with the α4β7-specific antibody (clone Act1). (PDF)

S7 Fig Distribution of CD4^+^ T cell subsets in different patient and control groups. (PDF)

S8 Fig Representative plots of CCR5^+^, HLA-DR^+^, PD-1^+^ and CD39^+^ cells. (PDF)

S9 Fig α4β7 is not correlated with activation, CD4 count or plasma viral load in HIV. (PDF)

S10 Fig Ratio of CCR9 and α4β7 is inverted in peripheral blood and gut of healthy individuals and patients with UC. (PDF)

S11 Fig Increased frequencies of α4β7^+^ CD4^+^ T cells after *in vitro* stimulation with bead-bound anti-CD3/CD28. (PDF)
