# Supplementary material for: Comparison of the integrin α4β7 expression pattern of memory T cell subsets in HIV infection and ulcerative colitis
Source: PLoS One. 2019 Jul 29;14(7):e0220008. doi: 10.1371/journal.pone.0220008 (PMC6663001; doi:10.1371/journal.pone.0220008)
Supplement: S1 Table — (PDF) [file pone.0220008.s001.pdf]

Identifier	PBMC LPL	Sample	Initial UC diagnosis	Medication	Clinical response to UC therapy	Severity of UC	Duration of VDZ-therapy (weeks)	Calprotectin (µg/g)	CRP (mg/l)	Clinical comment
UC 1	PBMC	baseline	1978	Prednisolone, Mesalazine	n.a.	intermediate	0	1880	17	no response to initial therapy, pancreatitis
	PBMC	follow-up	n.a.	Prednisolone, Vedolizumab	no	intermediate-severe	19	2730	7	switch to TNF-a
UC 2	PBMC	baseline	2013	Prednisolone	n.a.	mild-intermediate	0	n.a.	<5	patient initiated treatment stop
	PBMC	follow-up	n.a.	Vedolizumab	partial	mild	47	n.a.	<5	
UC 3	PBMC	baseline	2009	Budesonide	partial	mild-intermediate	0	n.a.	<5	
UC 4	PBMC	baseline	2009	Azathioprine, Valgangciclovir, Mesalazine, UDC	n.a.	severe	0	n.a.	34	st. p. CMV colitis, PSC
	PBMC	follow-up	n.a.	Azathioprine, UDC, Mesalazine, Vedolizumab	no	severe	57	n.a.	31	worsening of symptoms
UC 5	PBMC	follow-up	1997	Vedolizumab	yes	in remission	16	54,3	<5	stricture
UC 6	PBMC	baseline	2016	5-ASA, Infliximab, Valganciclovir	n.a.	intermediate	0	416	<5	st. p. CMV colitis
UC 7	PBMC	baseline	2012	Azathioprine, Adalimumab	n.a.	mild-intermediate	0	149	<5	PSC, colectomy 2014
UC 8	PBMC	baseline	2016	5-ASA, Budenoside,*	n.a.	mild-intermediate	0	87,3	<5	PSC, post-LTX*
	PBMC	follow-up	n.a.	Vedolizumab, *	yes	mild-intermediate	20		<5	
UC 9	PBMC	baseline	2013	Azathioprine, Prednisolone	n.a.	intermediate-severe	0	290	<5	
	PBMC	follow-up	n.a.	Mesalazine, Vedolizumab	yes	complete remission	39	19,8	<5	
UC 10	LPL	not applicable	2018	Prednisolone, Mesalazine	n.a.	mild	n.a.	n.a.	<5	
UC 11	LPL	not applicable	2018	none	n.a.	mild	n.a.	n.a.	<5	
UC 12	LPL	not applicable	2018	none	n.a.	complete remission	n.a.	n.a.	<5	PSC
UC 13	LPL	not applicable	2018	Mesalazine, Budenoside	n.a.	mild	n.a.	n.a.	6	PSC
UC 14	LPL	not applicable	2018	Adalimumab, Meslazine	n.a.	mild	n.a.	n.a.	n.a.	Budd–Chiari syndrome
UC 15	LPL	not applicable	2018	Ustekinumab	n.a.	mild	n.a.	n.a.	<5	

*Prednisolone, Tacrolimus, Mycophenolat, UDC

S1 Table: Cohort statistics of patients with ulcerative colitis. Baseline refers to VDZ-naïve patients, follow-up refers to patients that have received VDZ**.** PBMC, peripheral blood mononuclear cells**;** LPL, lamina propria lymphocytes; n.a., information not available.
