# Supplementary material for: Comparison of the integrin α4β7 expression pattern of memory T cell subsets in HIV infection and ulcerative colitis
Source: PLoS One. 2019 Jul 29;14(7):e0220008. doi: 10.1371/journal.pone.0220008 (PMC6663001; doi:10.1371/journal.pone.0220008)
Supplement: S1 Fig — (PDF) [file pone.0220008.s002.pdf]

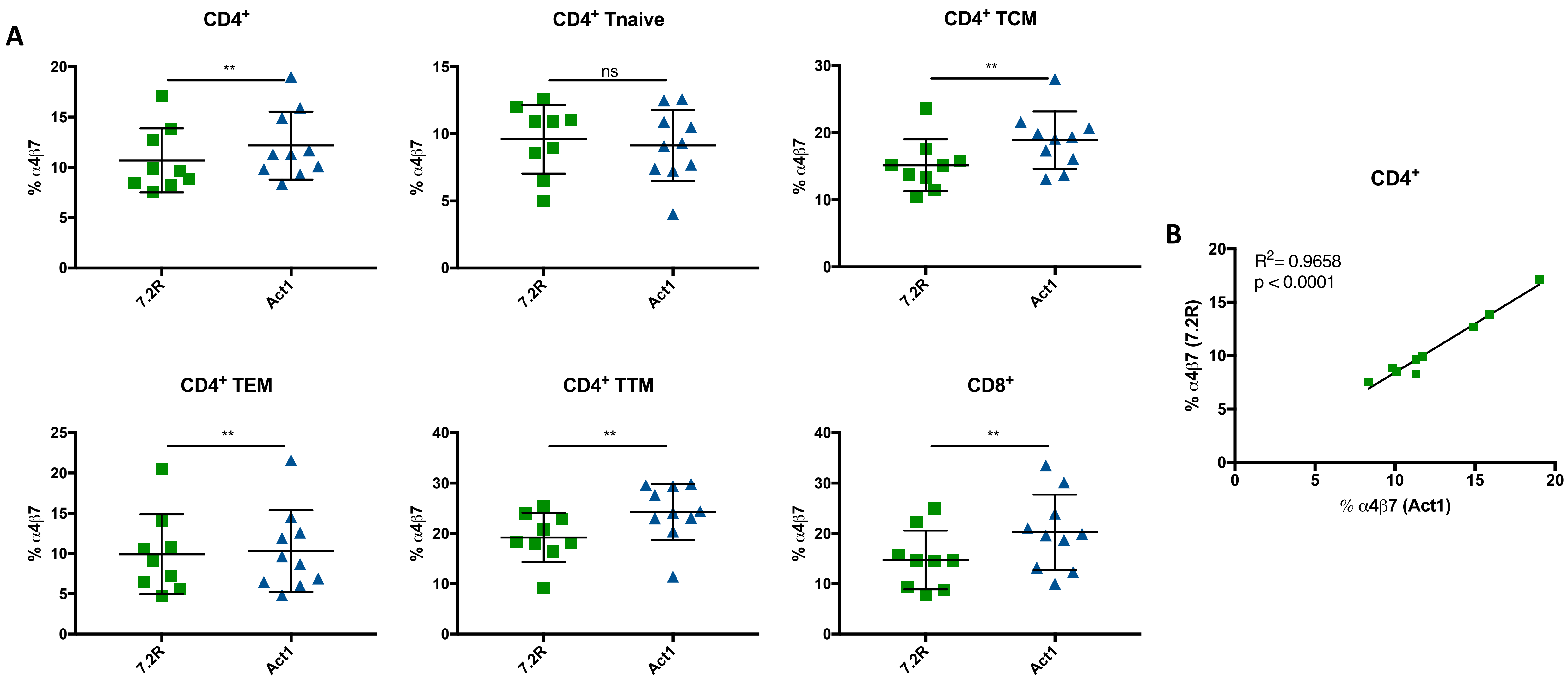

Supplemental Figure S1: Comparison of the α4β7-specific clone Act1 and the α4-specific clone 7.2R in combination with a separate β7-specific antibody.

A PBMC of healthy controls were stained with either an α4-specific antibody (clone 7.2R) in combination with a β7-specific antibody (clone FIB504) (left, green) or with the α4β7-specific antibody, clone Act1 (right, blue). Results were analysed using the Wilcoxon matched-pairs signed rank test, nonparametric test of paired samples. **B** Analysed frequencies of both staining approaches are highly correlated.
