# Supplementary material for: Comparison of the integrin α4β7 expression pattern of memory T cell subsets in HIV infection and ulcerative colitis
Source: PLoS One. 2019 Jul 29;14(7):e0220008. doi: 10.1371/journal.pone.0220008 (PMC6663001; doi:10.1371/journal.pone.0220008)
Supplement: S2 Fig — (PDF) [file pone.0220008.s003.pdf]

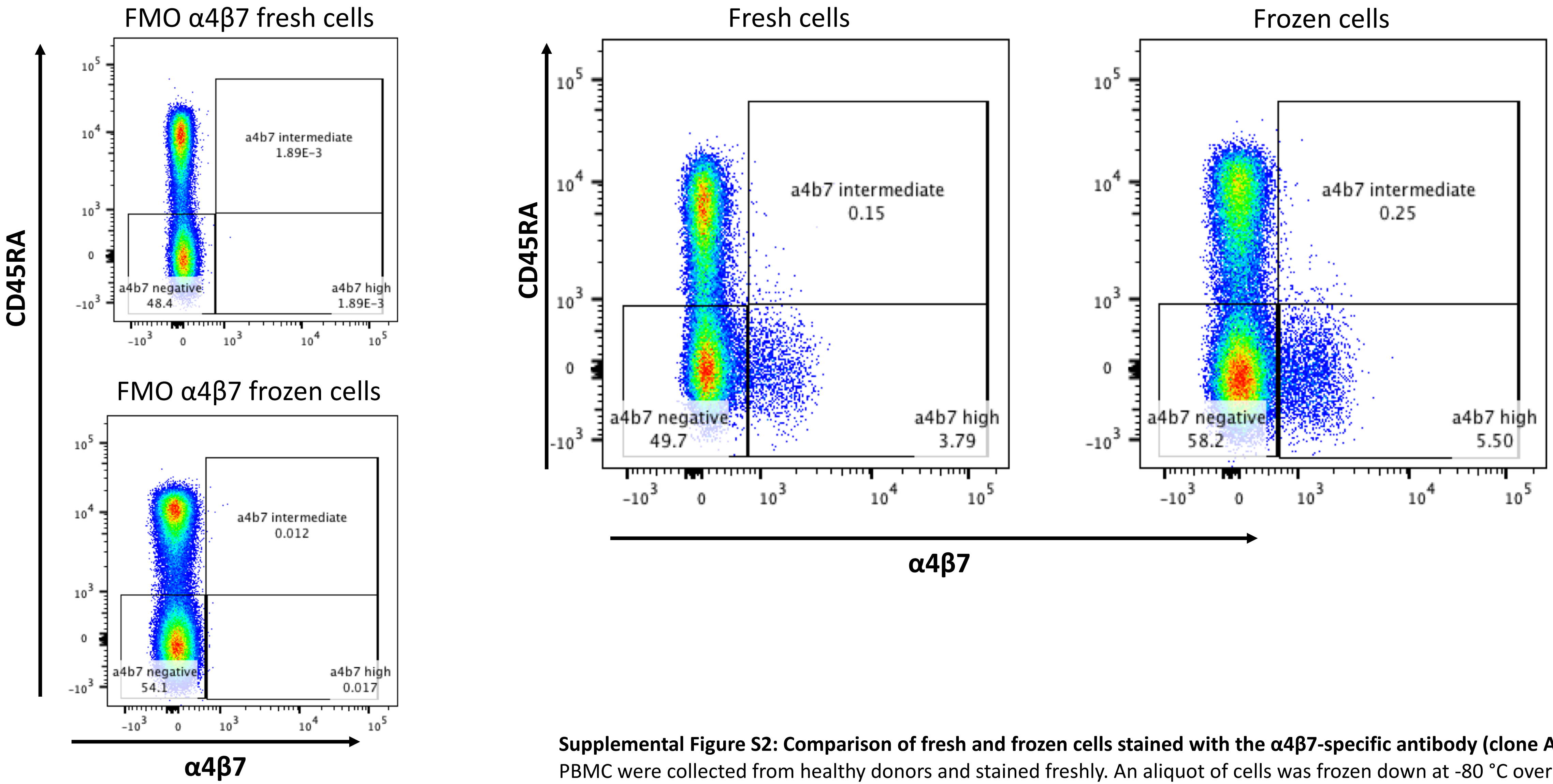

Supplemental Figure S2: Comparison of fresh and frozen cells stained with the α4β7-specific antibody (clone Act1).
PBMC were collected from healthy donors and stained freshly. An aliquot of cells was frozen down at -80 °C overnight and stained the following day. Gating according to fluorescence minus one (FMO) controls.
