# Supplementary material for: Comparison of the integrin α4β7 expression pattern of memory T cell subsets in HIV infection and ulcerative colitis
Source: PLoS One. 2019 Jul 29;14(7):e0220008. doi: 10.1371/journal.pone.0220008 (PMC6663001; doi:10.1371/journal.pone.0220008)
Supplement: S3 Fig — (PDF) [file pone.0220008.s004.pdf]

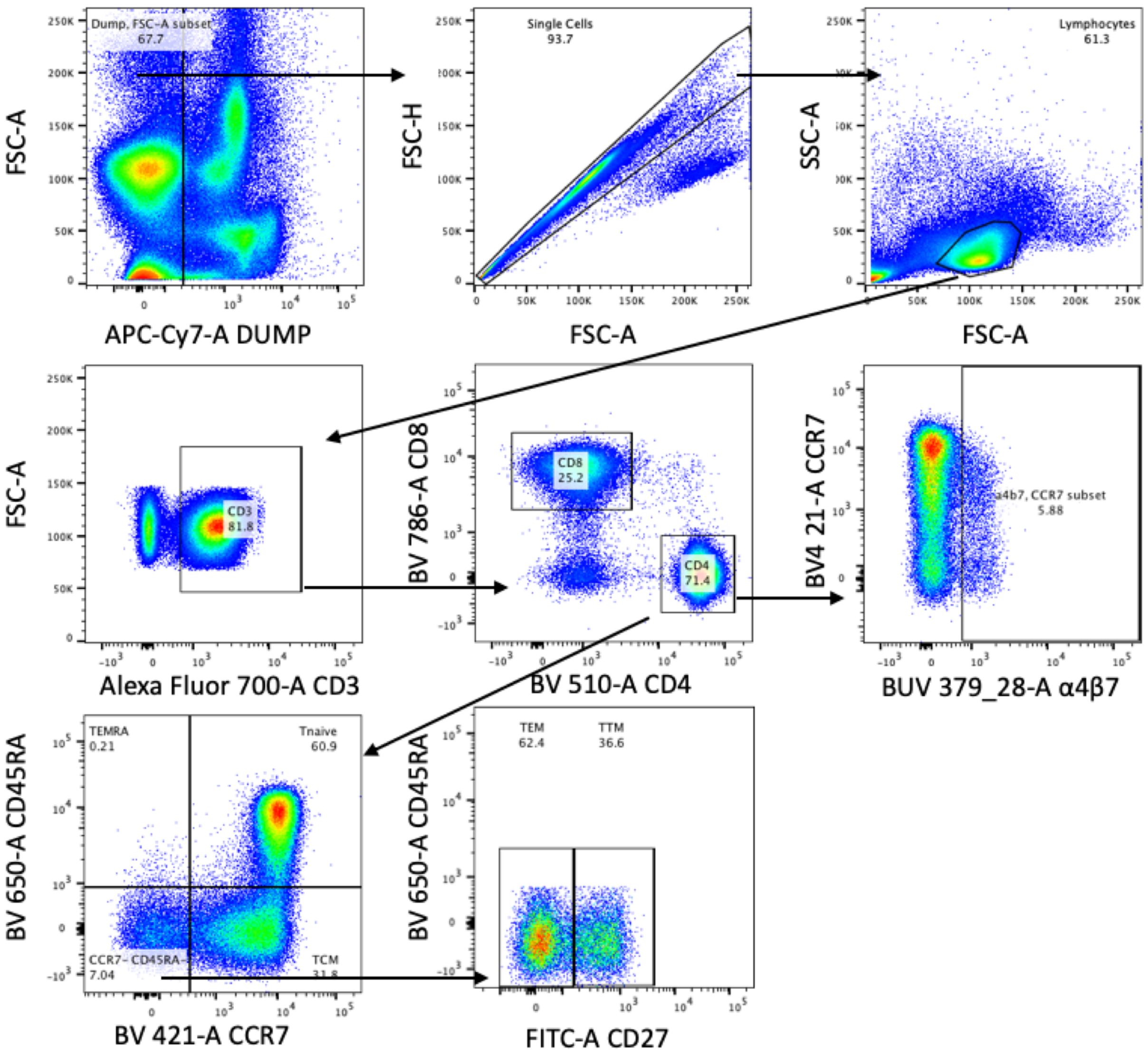

Supplemental Figure S3: Gating strategy for CD4⁺ α4β7⁺ T cells stained with an the α4β7-specific (clone Act1) antibody.
Live cells, single cells, lymphocytes, CD3⁺, CD4⁺ T cells, α4β7⁺ CD4⁺ T cells.
