# Supplementary material for: Comparison of the integrin α4β7 expression pattern of memory T cell subsets in HIV infection and ulcerative colitis
Source: PLoS One. 2019 Jul 29;14(7):e0220008. doi: 10.1371/journal.pone.0220008 (PMC6663001; doi:10.1371/journal.pone.0220008)
Supplement: S4 Fig — (PDF) [file pone.0220008.s005.pdf]

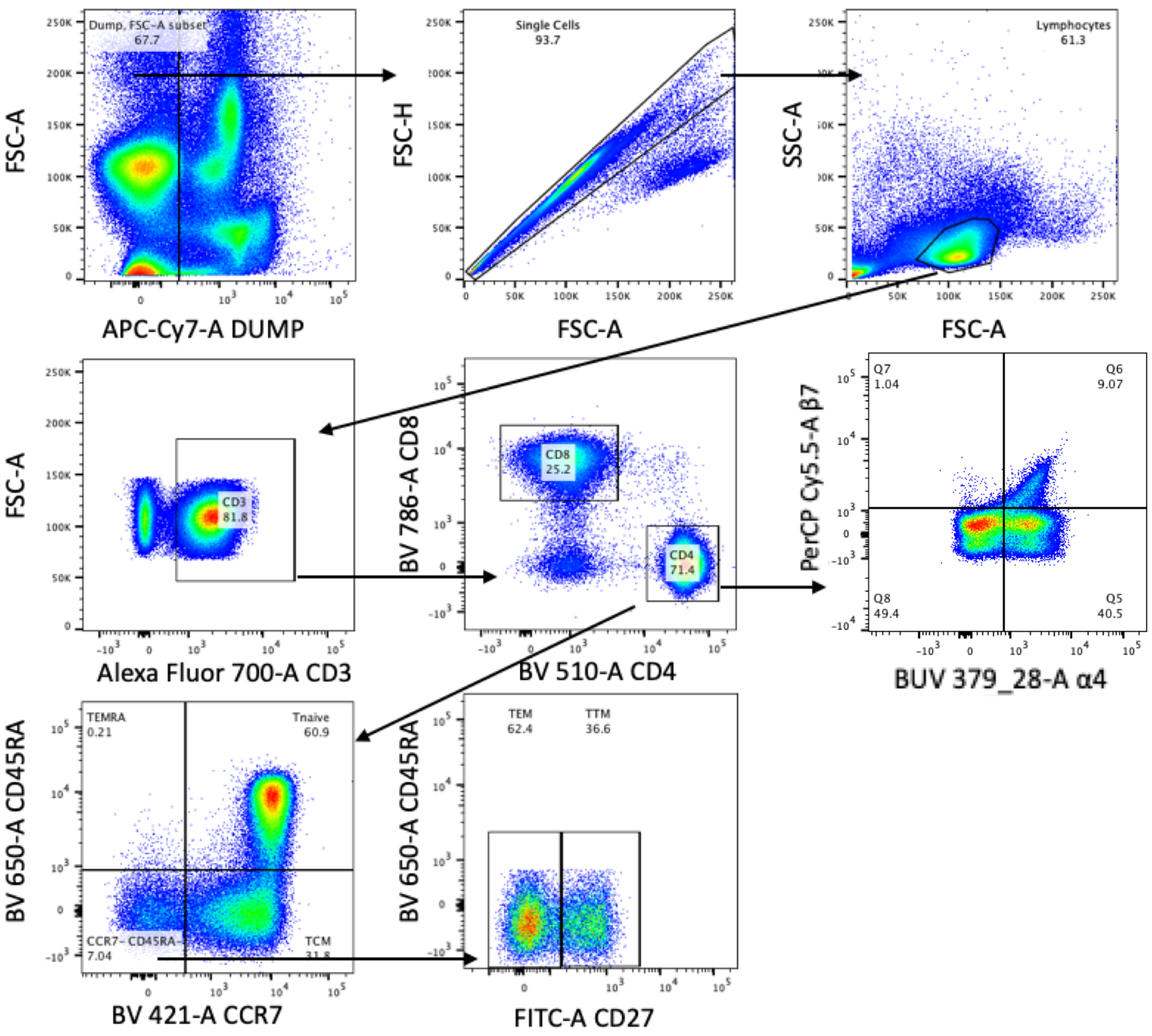

Supplemental Figure S4: Gating strategy for CD4^{+} α4β7^{+} T cells stained with an α4-specific (clone 7.2R) and a β7-specific (clone FIB504) antibody.
A Gating strategy: Live cells, single cells, lymphocytes, CD3^{+}, CD4^{+} T cells, α4β7^{+} CD4^{+} T cells (Q2), **B** Fluorescence minus one (FMO) controls used for gating of α4^{+}β7^{+} CD4^{+} T cells.
