# Supplementary material for: Comparison of the integrin α4β7 expression pattern of memory T cell subsets in HIV infection and ulcerative colitis
Source: PLoS One. 2019 Jul 29;14(7):e0220008. doi: 10.1371/journal.pone.0220008 (PMC6663001; doi:10.1371/journal.pone.0220008)
Supplement: S8 Fig — (PDF) [file pone.0220008.s009.pdf]

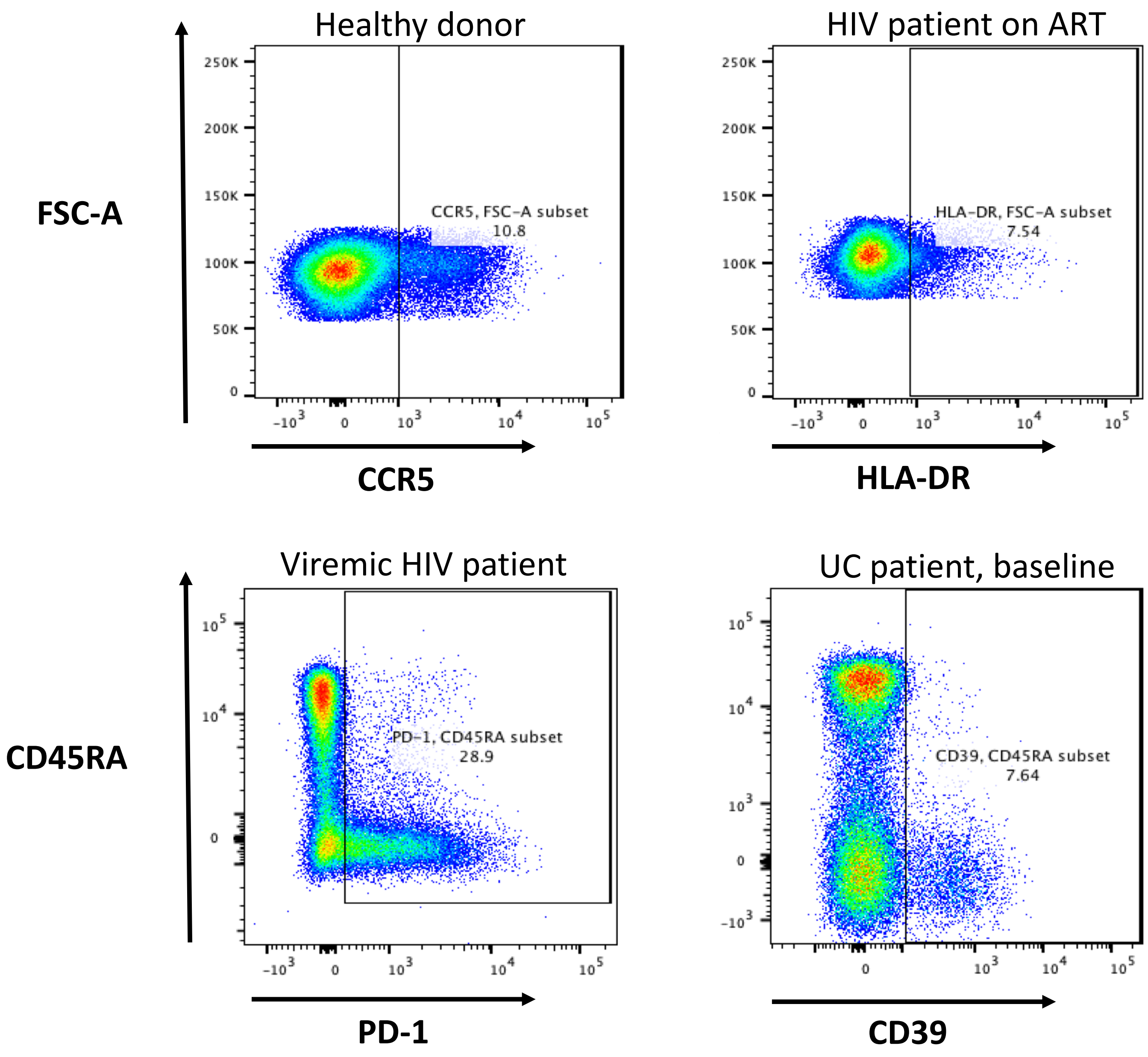

Supplemental Figure S8: Representative plots of CCR5⁺, HLA-DR⁺, PD-1⁺ and CD39⁺ cells.
