# Supplementary material for: Comparison of the integrin α4β7 expression pattern of memory T cell subsets in HIV infection and ulcerative colitis
Source: PLoS One. 2019 Jul 29;14(7):e0220008. doi: 10.1371/journal.pone.0220008 (PMC6663001; doi:10.1371/journal.pone.0220008)
Supplement: S9 Fig — (PDF) [file pone.0220008.s010.pdf]

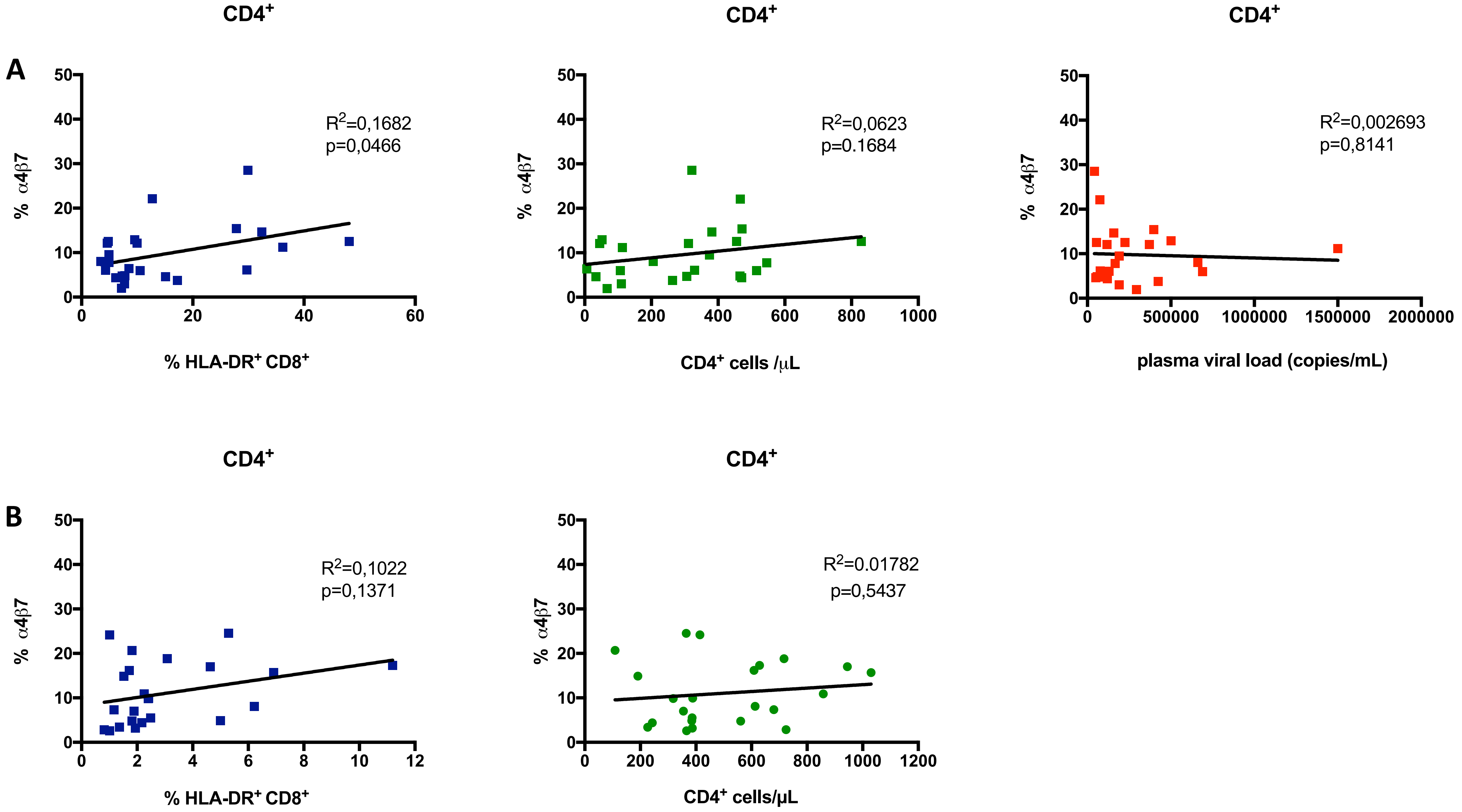

Supplemental Figure S9: α4β7 is not correlated with activation, CD4 count or plasma viral load in HIV. General activation is represented by HLA-DR$^+$ CD8$^+$ T cells. Representative plots are shown which depict total CD4$^+$ T cells. **A** viremic patients, **B** patients on ART.
