# Supplementary material for: Comparison of the integrin α4β7 expression pattern of memory T cell subsets in HIV infection and ulcerative colitis
Source: PLoS One. 2019 Jul 29;14(7):e0220008. doi: 10.1371/journal.pone.0220008 (PMC6663001; doi:10.1371/journal.pone.0220008)
Supplement: S10 Fig — (PDF) [file pone.0220008.s011.pdf]

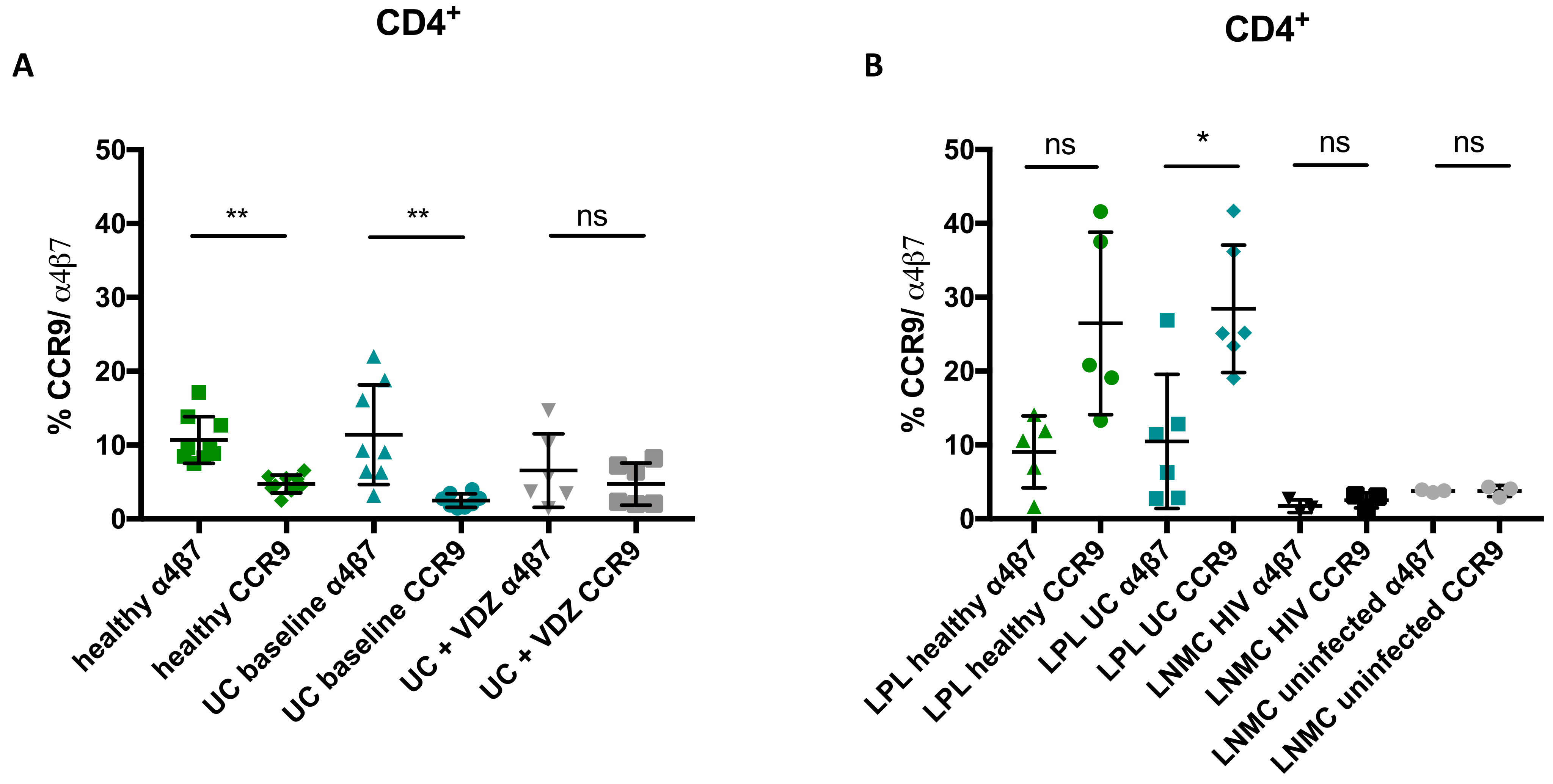

Supplemental Figure S10: Ratio of CCR9 and α4β7 is inverted in peripheral blood and gut of healthy individuals and patients with UC.

Frequencies of α4β7 and CCR9 on $CD4^+$ PBMC (A) and LPL and LNMC (B).UC, ulcerative colitis; LPL, lamina propria lymphocytes; LNMC, lymph node mononuclear cells; PBMC, peripheral blood mononuclear cells.
