# Supplementary material for: Comparison of the integrin α4β7 expression pattern of memory T cell subsets in HIV infection and ulcerative colitis
Source: PLoS One. 2019 Jul 29;14(7):e0220008. doi: 10.1371/journal.pone.0220008 (PMC6663001; doi:10.1371/journal.pone.0220008)
Supplement: S11 Fig — (PDF) [file pone.0220008.s012.pdf]

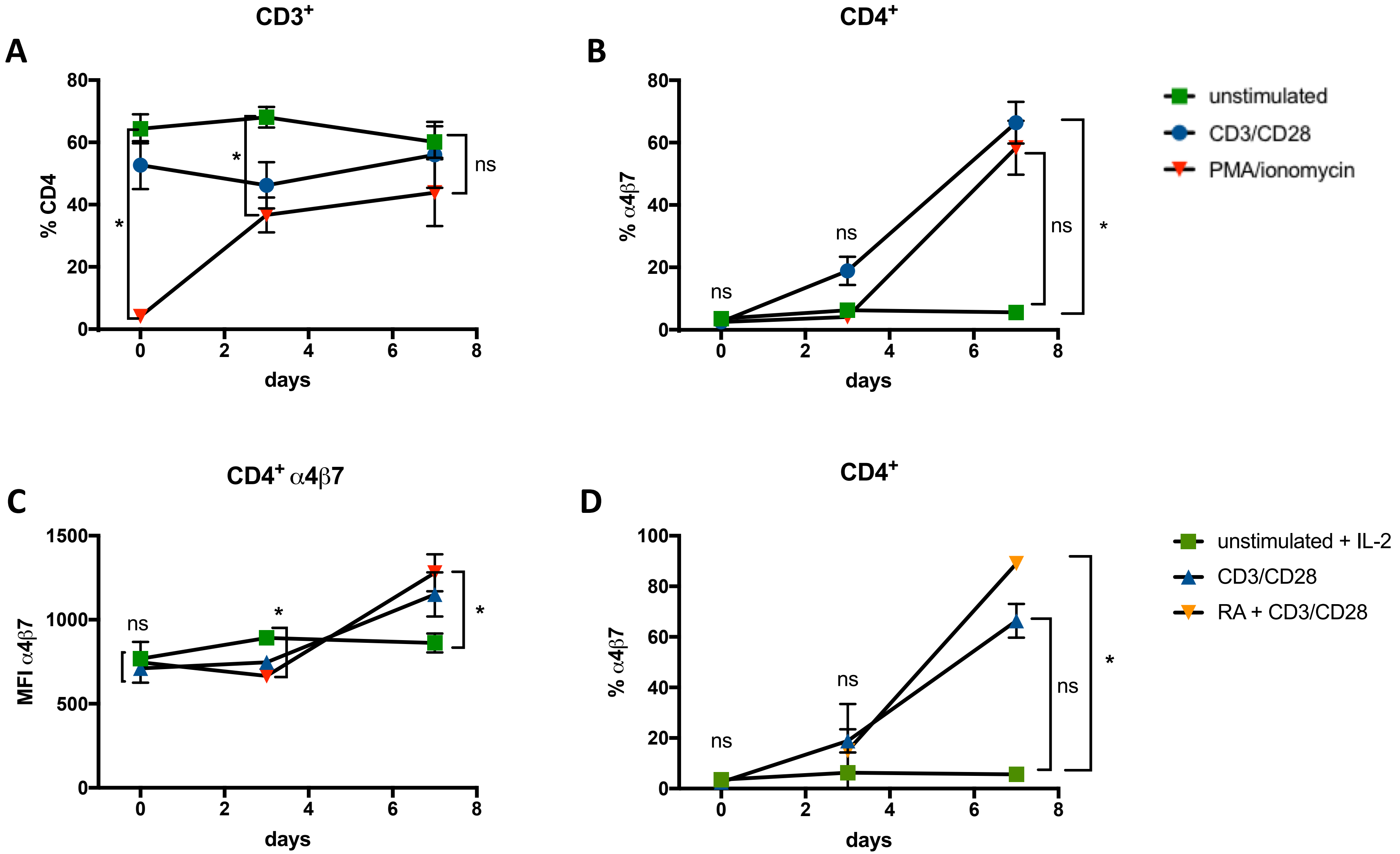

Supplemental Figure S11: Increased frequencies of α4β7⁺ CD4⁺ T cells after *in vitro* stimulation with bead-bound anti-CD3/CD28.

A Percentage of $CD4^+$ T cells. **B** Percentage of α4β7-expressing $CD4^+$ T cells. **C** Mean fluorescence intensity (MFI) of α4β7-expressing $CD4^+$ T cells. **D** Percentage of α4β7-expressing $CD4^+$ T cells after stimulation with retinoic acid (RA) and bead-bound CD3/CD28. Day 0 depicts the status of the cells after 6 hours of stimulation. Friedmann tests for multiple comparisons were performed.
